# Alignment-independent technique for 3D QSAR analysis

**DOI:** 10.1007/s10822-016-9909-0

**Published:** 2016-03-30

**Authors:** Jon G. Wilkes, Iva B. Stoyanova-Slavova, Dan A. Buzatu

**Affiliations:** Division of Systems Biology, National Center for Toxicological Research, 3900 NCTR Road, Jefferson, AR 72079 USA

**Keywords:** 3D modeling, Molecular conformation, Spectral data-activity relationship, Quantitative structure–activity relationship

## Abstract

Molecular biochemistry is controlled by 3D phenomena but structure–activity models based on 3D descriptors are infrequently used for large data sets because of the computational overhead for determining molecular conformations. A diverse dataset of 146 androgen receptor binders was used to investigate how different methods for defining molecular conformations affect the performance of 3D-quantitative spectral data activity relationship models. Molecular conformations tested: (1) global minimum of molecules’ potential energy surface; (2) alignment-to-templates using equal electronic and steric force field contributions; (3) alignment using contributions “Best-for-Each” template; (4) non-energy optimized, non-aligned (2D > 3D). Aggregate predictions from models were compared. Highest average coefficients of determination ranged from R_Test_^2^ = 0.56 to 0.61. The best model using 2D > 3D (imported directly from ChemSpider) produced R_Test_^2^ = 0.61. It was superior to energy-minimized and conformation-aligned models and was achieved in only 3–7 % of the time required using the other conformation strategies. Predictions averaged from models built on different conformations achieved a consensus R_Test_^2^ = 0.65. The best 2D > 3D model was analyzed for underlying structure–activity relationships. For the compound strongest binding to the androgen receptor, 10 substructural features contributing to binding were flagged. Utility of 2D > 3D was compared for two other activity endpoints, each modeling a medium sized data set. Results suggested that large scale, accurate predictions using 2D > 3D SDAR descriptors may be produced for interactions involving endocrine system nuclear receptors and other data sets in which strongest activities are produced by fairly inflexible substrates.

**Electronic supplementary material:**

The online version of this article (doi:10.1007/s10822-016-9909-0) contains supplementary material, which is available to authorized users.

## Introduction

3-Dimensional spectral data-activity relationship (3D-SDAR) modeling is a grid-based in silico technique which belongs to a group of methods collectively known as Structure–Activity Relationships (SARs). In 3D-SDAR each compound is represented by a unique “fingerprint” constructed from the NMR chemical shifts, δ, of all carbon atom pairs placed on the X- and Y-axes joined with the inter-atomic distances between each pair on the Z-axis [1]. For details see the sub-section below on the 3D-QSDAR fingerprint. The atom-specific nature of chemical shifts and the use of inter-atomic distances enable representation of interaction potential with receptor active sites in terms of electronic and steric qualities, respectively [2]. 3D-SDAR can produce models that facilitate identification of 3D pharmacophores and toxicophores.

This work models quantitative data and so exemplifies 3D-QSDAR. In this project, information about the presence of atoms other than carbon was not explicitly included. We have found in several of our previous SDAR modeling projects that the high sensitivity of ^13^C δs to their environment is often sufficient for useful reflection of chemical structure in the vicinity, including the presence of nearby heteroatoms [1, 3, 4]. A tessellation of the 3D-SDAR space into regular grids (“binning”) is further used to convert the information contained in a fingerprint into a set of 3D-SDAR descriptors. For a particular molecule, in addition to the 3D co-ordinates from carbon atoms, each descriptor includes the number of fingerprint elements belonging to each bin. Depending on the granularity of the grid, thousands of such descriptors can be generated but most bins have zero occupancy. These are further handled by an ensemble modeling PLS algorithm performing multiple training/hold-out test randomization cycles producing averaged “composite” models.

The 3D-SDAR parametric space, with a quantitative measure of each compound’s biological affinity appended, can be explored by comparing the predictive power of models derived from grids of different density/granularity, thus determining an optimal grid size. As a 3D-modeling technique conceptually similar to Comparative Molecular Field Analysis (CoMFA) and Comparative Molecular Similarity Analysis (CoMSIA), 3D-QSDAR depends on the specific conformation chosen for fingerprint generation. Unlike CoMFA and CoMSIA, 3D-QSDAR is an alignment independent technique.

Our earlier studies indicated that 3D-QSDAR models based on lowest energy conformations perform well [1, 3, 4]. However, we hypothesized that use of substrates internally aligned with respect to molecular template molecules rather than energy-minimized conformations might prove beneficial. In other words, we asked whether the adoption of a biologically more appropriate conformation for flexible compounds significantly increases overall predictive accuracy of the models by using 3D descriptors. Addressing this was the point of an experimental design intended to explore a variety of ways to establish 3D conformations. As will be shown below, the hypothesis was contradicted for modeling androgen receptor binding (the endpoint studied here) and another similar challenge.

Whether substrate-template alignment or energy minimization generates optimal 3D-SDAR models has not been previously determined. By definition of the 3D-SDAR fingerprints, substrate conformational changes would affect the position of the fingerprint elements only along the Z-axis. Because the distance between first and second order atom neighbors does not change with conformation, only fingerprint elements associated with more widely separated atom pairs in flexible molecules would vary significantly.

To study the effect of conformation on the performance of 3D-QSDAR models, the following experiments were conducted:

- conformational search analysis for each molecule to locate the global minimum of the potential energy surface (PES) followed by a semi-empirical or QM optimization to determine it precisely;
- alignment-to-template molecules [5], performed using clustering by similarity (alignment-to-templates by two different procedures was tested);
- simple 2D to 3D (2D > 3D) conversion using molecular mechanics as implemented in Jmol.

Approaches (a) and (b) guaranteed the use of consistent and reproducible geometries [6]; approach (c), though much less computationally demanding, was not systematic and models based thereon might not be precisely reproducible. If our hypothesis was true, approach (c) should produce inferior results compared to (a) or (b). These approaches were compared for predictive accuracy of the resulting 3D-QSDAR models.

Many conformational alignment algorithms have been developed for use in 3D-QSAR modeling [7–10]. Choices related to alignment are discussed in detail in Materials and Methods, the subsection entitled 3D-QSDAR Conformation Comparison, Experimental Design.

To test how a specific choice for generating conformations affects the predictive performance of 3D-QSDAR models, a dataset of 146 compounds, each with known affinity to the androgen receptor (AR), was used. The biological and environmental significance of modeling androgenicity is discussed in Online Resource 1, file name ESM_1 [11–17]. In summary, binding of exogenous chemicals to the AR leads to mammalian endocrine system disruption, which has happened and is happening on a large scale.

The dataset of 146 AR binders satisfied the following requirements: (1) they were all measured in the same lab using the same methods by the same personnel; (2) they were structurally diverse (>10 carbon backbone classes); (3) a significant proportion were flexible compounds (see “Discussion” of Kier Index below), (4) they involved interaction with a single, well defined biological receptor, and (5) the same data were previously used in QSAR modeling (for direct comparison of results).

We examined how use of different conformations affected the overall statistical accuracy of 3D-QSDAR predictions, the optimal bin dimensions, and/or the locations of important bins in 3D-SDAR space. We also studied whether consensus predictions averaged from models based on different molecular conformations would lead to increased predictive accuracy. A model based on directly downloaded (2D > 3D) structures without systematic conformational adjustment or alignment was also built and used to identify substructural elements that contribute to AR binding and endocrine system disruption. Finally, the general utility of the 2D > 3D shortcut was studied for two other biological endpoints by comparing model predictive accuracy based on 2D > 3D conformations to those based on energy minimized conformations.

These studies have been successfully completed and may lead to a significant improvement in computational modeling: a methodology that uses 3D descriptors for which it will be practical to predict biological affinity accurately for a huge chemical data set. This capability becomes possible by avoiding computationally-intensive and subjective procedures necessary for other 3D methods to build and consult models.

## Materials and methods

### Data set

146 androgen receptor binders from the Nationalm Center for Toxicological Researh (NCTR) Endocrine Disruption Knowledge Base (EDKB) were used along with their respective binding affinities as a representative and illustrative modeling challenge (http://www.fda.gov/scienceresearch/bioinformaticstools/endocrinedisruptorknowledgebase/default.htm). Experimental Relative Binding Affinities (RBA) to AR were determined by measuring the binding inhibition of radiolabeled [^3^H] R1881 to the rat androgen receptor. There were far more of the less active compounds than of the more active in this data set. To improve data normality, logarithms of RBA were used for modeling [18].

Structure or chemical utility classes among the 146 included steroids, DESs, DDTs, flutamides, indoles, PCBs, pesticides, phenols, phthalates, phytoandrogens, and siloxanes. The study varied the basis for defining 3D conformations. The range of possible conformations was obviously related to the inherent flexibility of molecules in the data set. Some of the structures were not very flexible.

Each of the compounds was analyzed for structural flexibility using the Kier Index of Molecular Flexibility [19]. The Kier Index is a dimensionless indicator of relative flexibility. While it yields a quantitative value, the meaning is more intuitive, qualitative. A completely flexible molecule, such as a very long alkane chain, would have an infinite Kier Index. The 146 compounds in this study range from about 1.7 to 14.4 on the index. 48 (32.9 %) of the compounds have indices below 3.0 and could be described as fairly rigid. 70 (47.9 %) have indices between 3.0 and 5.0 and could be described as partially or somewhat flexible. The remaining 28 (19.2 %) molecules have the higher indices and would be described as flexible.

Each chemical used in this study along with its conformational alignment template, CAS number, log(RBA) to AR, and 2D structure drawing is catalogued in Online Resource 2, file name ESM_2. Online Resource 3 reports molecule-specific results of the Kier Index calculation.

### Definition of terms, description of computational tools, and basic concepts

PLS was used for model generation. SDAR and many other descriptor sets provide a large number of variables. Multiple Linear Regression (MLR) fails if the number of variables exceeds the number of data entries (compounds, here). PLS reduces the dimensionality so that the pattern is encoded as weighted contributions from the original variables (bins, in SDAR). Most of the variability in the data is condensed into the first few, orthogonal Latent Variables (LVs). The mathematical operation that generates the LVs is reversible via the model weights in each LV, so that the contributions of bins to the model are interpretable.

Non-linear modeling techniques can be interpreted to some extent but the process is not easy and the results are qualitative [20]. It is difficult via their non-linear associations to relate the molecular descriptors to the observed binding affinity and thus develop structure–activity associations from the model. Because SDAR descriptors are directly related to chemical structure, and PLS models can be used to identify important bins, the combination of SDAR with PLS facilitates discovery of pharmacophores or toxicophores.

NMR chemical shifts and many interatomic distances exist along a continuum. Combined to form 3D fingerprints, the ordered triplets are binned so that elements belonging to the same bin (and presumptively residing in similar chemical environments) are likely to contribute in a similar manner to the biological affinity. Since we did not know a priori the optimal grid granularity, bin widths ranging from 2 to 20 ppm in the XY chemical shift plane were explored in 2 ppm increments. Beyond 20 ppm, atoms in quite different chemical environments would be grouped together, thus reducing the ability to infer structural associations from the models. On the Z-axis, the interatomic distances were binned varying from 0.5 to 2.5 Å in 0.5 Å increments. Beyond 2.5 Å, atoms across a phenyl ring from each other, for example, might meet the same criteria in relation to a distant atom, so that structural alert discovery would become more difficult. Systematically examining all possible combinations of bin granularity necessitated batch-mode operations, here performed automatically by algorithms written in Matlab R2012b.

To reduce error, achieve reproducibility, assure objectivity, and avoid data over-fitting, a PLS modeling algorithm employed a random number generator (RNG) to create a sequence of training and test set combinations. A batch of 100 randomization cycles executed at each bin width granularity of the SDAR grid was performed. In each randomization event, 20 % of the compounds was held out as a test set, the remaining 80 % was used to build the model and to predict the binding affinity of the held out compounds. It has been demonstrated that randomization rules that disregard applicability domains, as in this case, produce more conservative estimates of the external predictive performance of models [21]. This approach was adopted in our experimental design. After 100 iterations, statistical metrics (R_Training_^2^, R_Test_^2^, R_Scrambling_^2^) were calculated as averages of the corresponding values in each cycle. The RNG was automatically reset to the same seed for the next experiment in a batch. Systematic examination of parameter space in batch operation yielded 20 % hold-out test set predictions from which R_Test_^2^ values were calculated. In other 3D-SDAR projects, R_Test_^2^ results thus obtained have shown accuracy equivalent to that for predicting toxicity for members of an external test set [22]. Predictions were also generated for training set compounds as well as for test set compounds based on Y-scrambled inputs. In the latter case, the list of binding affinities were randomly distributed among the 146 compounds to assess the likelihood that nonsense relationships would be “discovered.”

*Composite* models were averaged from predictions of 100 individual models. Predictions from a composite model could be further averaged with those of other composite models developed either under different modeling parameters or based upon different 3D molecular conformations, thus generating a *consensus* model.

### The 3D-QSDAR “fingerprint”

Organic molecules with at least two carbon atoms can be represented by the 3D spectral fingerprints used here [1]. For a given molecule with a total of *N* ≥ 2 carbon atoms, the 3D fingerprint is constructed using the chemical shifts of all non-ordered (C_i_C_j_ ≡ C_j_C_i_; i, j = 1, …, N) carbon atom pairs in conjunction with a δC_i_ ≥ δC_j_ condition, in which δ denotes a chemical shift in ppm. Under these conditions, a 3D abstract space is created having the following orthogonal axes: (1) the δ of atom C_i_ is placed on the X-axis; (2) the δ of atom C_j_ is placed on the Y-axis, and (3) the distance (r_ij_) between atoms C_i_ and C_j_ forms the Z-axis. According to the above definition of axes, (C_i_C_j_ ≡ C_j_C_i_), all fingerprints are characterized by a single plane of symmetry C_s_ intersecting the XY-plane through its main diagonal. Application of δC_i_ ≥ δC_j_ removes the redundant fingerprint elements on one side of the symmetry plane.

These fingerprints are invariant under rotation and/or translation of the molecular atomic Cartesian coordinates. Thus, 3D-SDAR and 3D-QSDAR can be performed without conformational alignment, a significant advantage compared to CoMFA and CoMSIA. This explains why, prior to this work, alignment was not tested for 3D-QSDAR.

### 3D-QSDAR conformational comparison, experimental design

We compared the performance of 3D-QSDAR models based on four molecular geometries: Global Minimum Energy; Alignment-to-a-Template-50:50, (i.e., with equal contribution of the electronic and steric energies of interaction); Alignment-to-a-Template-Best-of-Each, (i.e., with optimized electronic and steric field contributions specific for each template), and a 2D to 3D conversion using instant JChem with an MM universal force field as implemented in ChemSpider, shorthand referenced as “2D > 3D”). These terms require more definition, explanation, and context.

For Global Minimum Energy models, the potential energy of each of the 146 molecules was determined by random walks followed by AM1 in Hyperchem 8.0 (HyperCube, Inc., Gainesville, FL) as detailed in Online Resource 4. When the difference in energy between consecutive AM1 iterations fell below 0.01 kcal/Å × mol, the calculation was at its convergence limit, computation was halted, and the associated molecular conformation was regarded as that with Global Minimum Energy. Each molecule’s Global Minimum Energy conformation was saved as a *.mol file and used to compute interatomic distances between pairs of carbon atoms. The distances and ^13^C NMR chemical shifts were combined as detailed below to define each molecule’s 3D-SDAR matrix, its fingerprint. After adding the log(RBA) to each fingerprint, the matrices were then used for PLS modeling.

Alignment-to-a-Template models presented another challenge. Due to the heterogeneity of the dataset and because binding profiles might not be the same for different structural groups, multiple templates were used. Many conformational alignment algorithms have been developed for use in 3D-QSAR modeling [7]. A commonly used approach is to define a group of structurally dissimilar templates that are active compounds and use force fields to align less active, flexible molecules to their most topologically similar templates. For alignment-to-template studies, we used force fit [8, 10]. Force field fitting procedures as implemented in Discovery Studio v 3.5 (BS Biovia, http://accelrys.com/) were applied to each flexible molecule such that its active features were positioned as close as possible to the corresponding features of its best template.

Obviously, template choice for each molecule was important [23]. Template for a molecule were based on its structural (carbon backbone) similarity to a molecule with strong or medium binding affinity to AR. We selected the strongest AR binder among molecules with a similar backbone in the data set to serve as that group’s template. This produced ten templates in all: 4-hydroxybiphenyl; 2-(4-nitrobenzyl)-1H-isoindole-1,3(2H)-dione; 6-hydroxyflavanone; dihydroxymethoxychlor olefin; dihydrotestosterone; *p*-nonylphenol; 4-hydroxybiphenyl; 4-hydroxy-tamoxifen; di-*n*-butyl phthalate; α-zearalenol; triphenyl phosphate. The CAS numbers of these ten compounds are catalogued in Online Resource 2. The plan was that template molecule conformations would be determined by X-ray crystallography as bound in the AR or, if that receptor data was not available, a similarly shaped nuclear receptor, the estrogen receptor (ER) [24]. Should neither be available, the template’s lowest energy unbound conformation would be used. The only bound conformations of template molecules available were for 4-hydroxy-tamoxifen in the estrogen receptor alpha (human) and dihydrotestosterone in the androgen receptor (rat). These two receptor-bound compound conformations were templates used for 11 and 43 compounds, respectively. This left 82 other compounds referenced to their template’s lowest energy conformation or, if completely inflexible, their only possible conformation.

See the table in Online Resource 2 for specific templates associated with each molecule. Only 27 compounds could not be assigned a template by visual inspection. Five of these were so structurally rigid that alignment was unnecessary, identified in the table by the annotation (N/A). 22 were flexible but so structurally distinct that it was not obvious which of the ten strong or medium AR-binding compounds could best serve as each ones template. For each the best template was selected based on a structural similarity index calculated using ToxMatch 1.0.7 [25]. Specifics of this process can be found in Online Resource 5 and Online Resource 6.

Genistein in comparison with its manually chosen template, 6-hydroxyflavanone, was arbitrarily chosen to scale expectations for ToxMatch template selections. Genistein’s ToxMatch similarity to 6-hydroxyflavanone was 0.63. The 22 flexible compounds’ similarity to their ToxMatch-selected templates ranged from 0.14 to 0.88, on average 0.39 ± 0.21. Average similarity less than that of the genistein, 6-hydroxyflavanone pair was not surprising, given the structural diversity that explained why the appropriate template for these 22 compounds was not obvious. Calculation of structural similarity provided objectivity in template selection. 2D structures of ToxMatch-defined pairs were also compared to confirm that their ToxMatch pairings appeared reasonable.

Another Discovery Studio feature allows the user to specify the relative contributions of electronic and steric force fields that determine optimal alignment. The program outputs a metric called Overlay Similarity: values range from −1 to +1, with +1 representing perfect alignment. The default relative contributions of electronic and steric fields are equal, 50:50. Starting with the Global Minimum Energy conformations, a new set of conformations for each of the 146 compounds was determined by choosing 50:50, and executing alignment to corresponding templates without regard to the resulting Overlay Similarity. The 50:50 aligned conformation for each molecule was written as a *.mol file and combined (See sub-section immediately below) with both the predicted NMR chemical shifts and experimental log(RBA)s. The set of such 3D-QSDAR matrices was modeled, yielding results labeled Alignment to-a-Template, 50:50.

For a third experiment, all compounds assigned to a particular template, were grouped together into a single *.sdf file (multiple *.mol files) and their Global Minimum Energy conformations were adjusted as a group relative to their template. Using 10 % increments, the relative contribution fraction was explored to identify that fraction (e.g., 100:0, 90:10, … 50:50, …10:90, 0:100) yielding the highest Overlay Similarity as a group to its template. The individual *.mol files were then extracted from the *.sdf files. 70:30 electrostatic:steric alignment was found optimal for each of the template groups. For this alternative alignment experiment, the 70:30 aligned conformation was used to define each molecule’s 3D-SDAR fingerprint. The set of fingerprints with appended experimental log(RBA)s was modeled and results were reported as Alignment-to-a-Template, Best-of-Each, since the experimental design concept was to use the force field proportions that produced the best fit for each template and the fact that each of these optimized at 70:30 was a coincidence.

Finally, we downloaded the 3D conformations from ChemSpider without conformational adjustments beyond what is done automatically by ChemSpider during the download of any 3D *.mol file: Jmol (Bioinformatics.Org) is used for 2D to 3D conversion by means of a molecular mechanics (MM) universal force field (UFF). We combined these mol files with their corresponding NMR files to form the 3D-SDAR fingerprint, added the log(RBA)s, performed the data set partitions and PLS analyses and reported results as 2D > 3D (meaning directly downloaded original conformations, non-systematically-energy minimized, non-aligned).

### Predicting ^13^C NMR chemical shifts, generating the 3D-QSDAR matrix, and optimizing models

The *.mol files were imported to the ACD/NMR C Predictor, Version 12.0, with the atom numbering system preserved in the transfer, and the NMR spectra of the corresponding compounds were generated using the HOSE algorithm [26, 27]. HOSE predicts on a two dimensional basis, that of the substructural unit. Three dimensional descriptor information used in 3D-SDAR and 3D-QSDAR derives from the combination of these 2D-predicted atomic chemical shifts with interatomic distances calculated from 3D mol files.

In a systematic examination of parameter space, the 3D-SDAR fingerprints were tessellated (binned) using regular grids. A step of 2 ppm was used to increment chemical shifts whereas a step of 0.5Å was used to increment interatomic distances…(i.e., 3D-SDAR fingerprints were tessellated using bins ranging in size from 0.5 Å × 2 ppm × 2 ppm to 2.5 Å × 20 ppm × 20 ppm). The use of small bin sizes increases the proportion of zero occupancy bins for the entire set of compounds. It also defines as distinct some adjacent chemical shift-distance combinations that actually might represent the same contribution to biochemical activity. The use of very large bins decreases the proportion of zero occupancy among the small number of large bins. But large bins implies, as being equivalent, chemical shift-distance combinations that might represent unrelated chemical effects, thus compromising the ability to infer associations. Therefore, for both quality-of-fit and pattern interpretation it was necessary to explore the granularity of parameter space using a multifactorial experimental design.

We explored model quality as a function of the number of PLS Latent Variables used in modeling as well as bin widths in both chemical shift and interatomic distance dimensions. For each compound and bin dimension combination, the number of fingerprint elements in each bin (the bin occupancy) was counted and stored in columns.

The binned data was processed using PLS and modeling results were subject to regression, generating R^2^ values. The R_Test_^2^ response space was compared, not the quality of training set results. The four modeling projects using from one to 10 LVs compared average R_Training_^2^ and R_Test_^2^ from parallel 100 fold random training/20 %-hold-uut test experiments. The addition of LVs stopped at the point at which R_Training_^2^ vs number of LVs has plateaued, while R_Test_^2^ and R_Scrambling_^2^ were monitored to ensure that the models were not produced due to chance and that their predictive power did not degrade significantly in comparison to the training set…

In the last 2 years our group has been using this rather stringent 20 %-Hold-Out and 100 random partitions validation process as standard procedure for quality assurance [1, 3, 4, 22]. This standard has recently been evaluated by others and confirmed as both necessary and appropriate for estimating QSAR model predictive accuracy of external data [21]. R_Training_^2^, R_Scrambling_^2^, and R_Test_^2^ are reported as average predictions from these 100 run cycles. This project entailed generating over 40,000 individual models and associated regressions.

### Consensus models

Besides comparing model statistics, we examined whether the different molecular conformation types had parallel or contrary error tendencies in prediction of log(RBA). If the error tendencies were contrary, predictions could benefit from consensus.

Since the RNG sequence of partitions was repeated exactly for each test, the average predictions could be directly compared across the experimental variable space to see whether there were differences in error tendencies between models based on different conformation types. For the four different conformation types we separately ranked the individual R_Test_^2^ values from greatest to least to see whether variations in conformation produced different sequences. If the identity of the training and test set compounds for a partition were the only significant factor, the rank would not differ with conformation. If outliers differed with conformation, ranking sequence would vary and consensus models from different conformations could show improved prediction accuracy.

### Discovering structure alerts of affinity or toxicity from 3D-SDAR models

We identified and plotted important bins from a 3D-SDAR model onto a 3D map of QSDAR abstract space. Some of the most important bins were plotted on the 3D-SDAR map, color coded to reflect the frequency of occurrence.

Important bins were used to discover structure alerts of toxicity: i.e., toxicophores. The task was to identify structural features that gave rise to atom pairs populating an important bin. Each such bin was manually overlaid on a chemical structure, each overlay appearing as a dotted, dashed, and/or colored line joining that bin’s pair of atoms. Once multiple overlays were constructed for several strongly interacting molecules, it was possible to discover structural features necessary to produce the binding affinity or, conversely for undesired affinity, features to avoid.

### Comparing results based on 2D > 3D versus energy optimized conformations

We built and modeled 2D > 3D conformations for 130 estrogens and 154 acute toxicity compounds previously modeled by 3D-QSDAR [1, 4]. Predictive accuracy, R_Test_^2^, was compared for these endpoints to estimate the circumstances under which one might effectively take advantage of the 2D > 3D shortcut and save 93–97 % of modeling time.

## Results

We report below results for models in which the experimental variations were explicitly designed to produce an objective comparison and not to bias for a particular conclusion. When it became obvious that the direct 2D > 3D conformations as applied to 3D-SDAR descriptors were outperforming conformation determinations requiring more elaborate procedures, an effort was made to understand whether this was an outlier result. Also, some experimental variants associated with template alignment models were explored to increase confidence that improved 2D > 3D performance was not an artifact of comparison to an inadequately optimized alignment procedure.

**Fig. 1:**
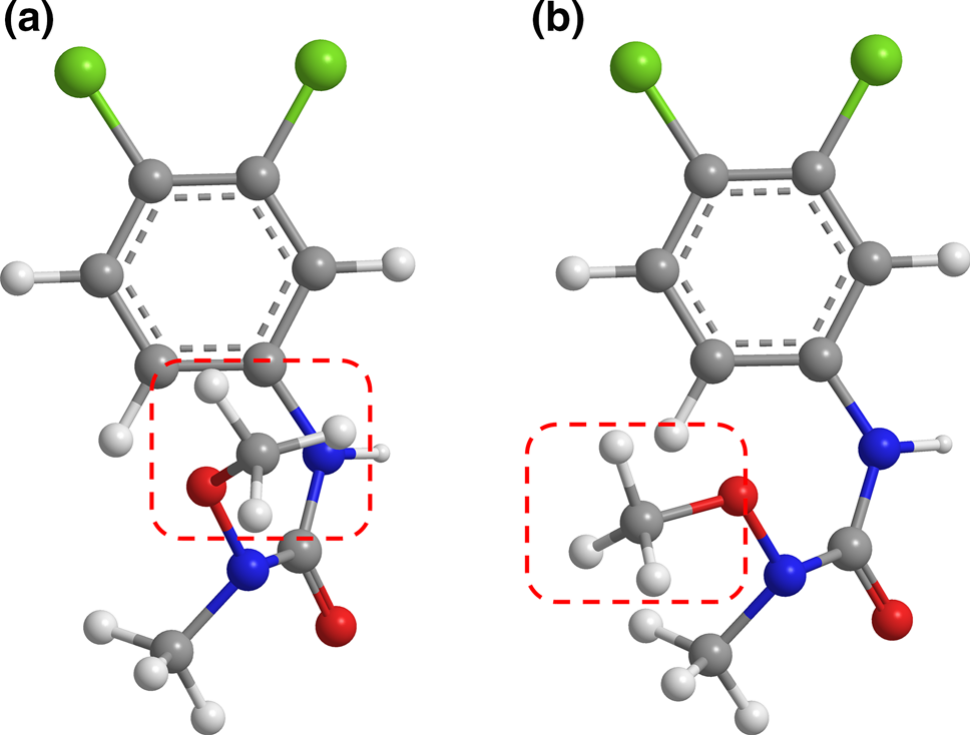
Example of how molecular conformation can vary with the method by which it is determined. Shown is linuron (Compound 106), in a 3D conformation based on **a** its internal Global Minimum Energy or **b** alignment to template 2-(4-nitrobenzyl)-1H-isoindole-1,3(2H)-dione (Compound 3) using 50:50 electronic:steric force fields. Orientation of a methyl group (inside the *red dashed box*) is the most obvious difference between the two conformations. The variation would be expressed in the abstract 3D SDAR fingerprint as a difference in the interatomic distance between the methyl carbon and other carbons in the molecule, which would affect Linuron’s SDAR fingerprint and could alter its predicted activity. The Kier Flexibility Index for Linuron is 4.55, which translates as “partially flexible”

We start by illustrating the relationship between molecular conformation and the SDAR fingerprint: the reason conformation would be expected to matter. Figure 1 visualizes a partially flexible molecule, Linuron. Its Global Minimum Energy conformation is shown in (a) and its conformation when aligned based on a 50:50 electronic:steric criterion, in (b). The most obvious conformational difference is highlighted.

### Predictive accuracy comparative statistics and response surfaces for AR models

Energy minimization and/or alignment affects the 3D conformation of non-rigid members among the 146 compounds, which might affect model accuracy. Detailed statistical results are available in Online Resources 4, 5, 6, and 7: Excel spreadsheets for Global Energy Minimized, Aligned 50:50, Aligned Best-of-Each 70:30, and direct 2D > 3D Conversion, respectively.

For Global Minimum Energy conformations, optimal predictive PLS models were built using 2–4 LVs. Two that showed good results:

Model (1): 4 LVs, average R_Test_^2^ = 0.60 for chemical shift bin width = 16 ppm and distance bin width = 1.0 Å (so bin dimensions in the abstract 3D- space were 16 ppm; 16 ppm; 1.0 Å)
Model (2): 3 LVs, average R_Test_^2^ = 0.60 for bin widths = 8 ppm and 1.0 Å (8 ppm; 8 ppm; 1.0 Å)

The Matlab code used in automatically building and holdout testing all models is provided in Online Resource 8, ESM_8.

**Fig. 2:**
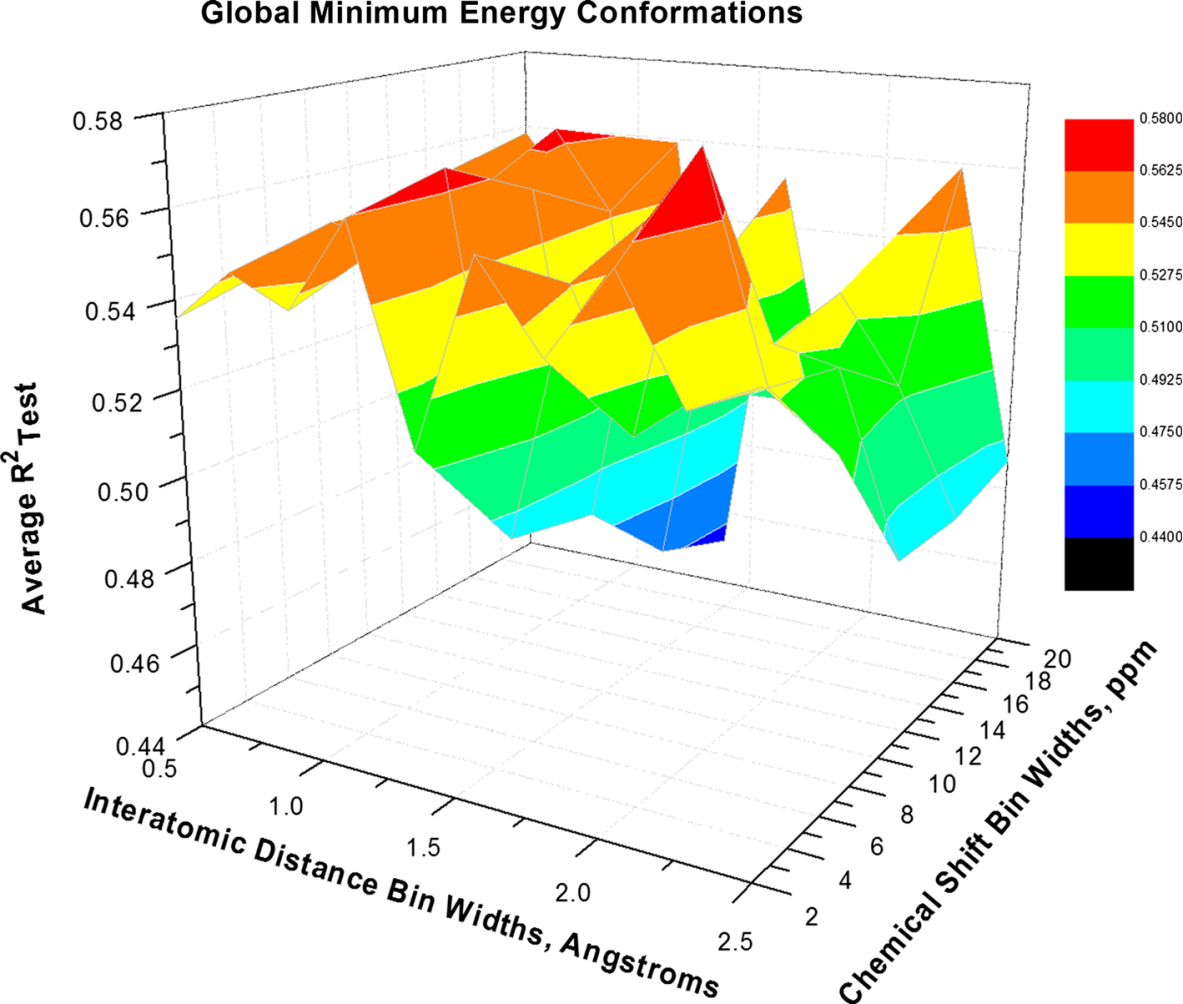
Response surface for Global Minimum Energy conformations modeled using 4 PLS Latent Variables (LVs). Plot shows average R_Test_^2^ as a function of chemical shift and interatomic distance bin widths. Three optima (surface regions *colored red*) span a range from 0.5 to 1.5 Å in interatomic distance granularity

**Fig. 3:**
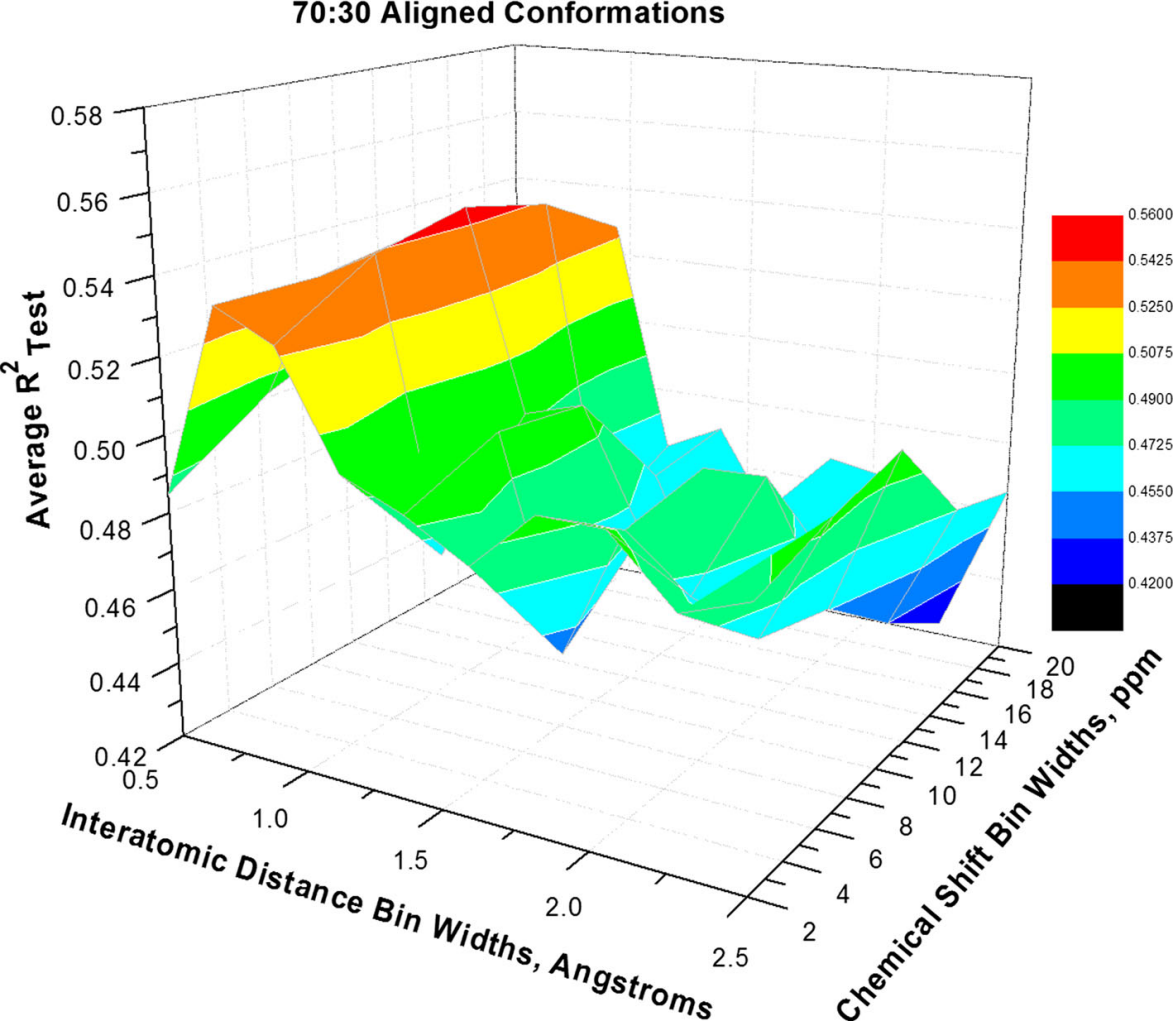
Average R_Test_^2^ response surface based on 70:30 Best-of-Each Alignment and 2 LVs. The surface shows a single optimum, indicated in *red*, though the *color key* in this case is translated downward by 0.02 R_Test_^2^ units compared to Fig. 2. The entire response surface is depressed on the *right side* there are no local optima found for large bins with 2.0–2.5 Å granularity. The response surface for 50:50 alignment was similar in shape to Best-of-Each Alignment

**Fig. 4:**
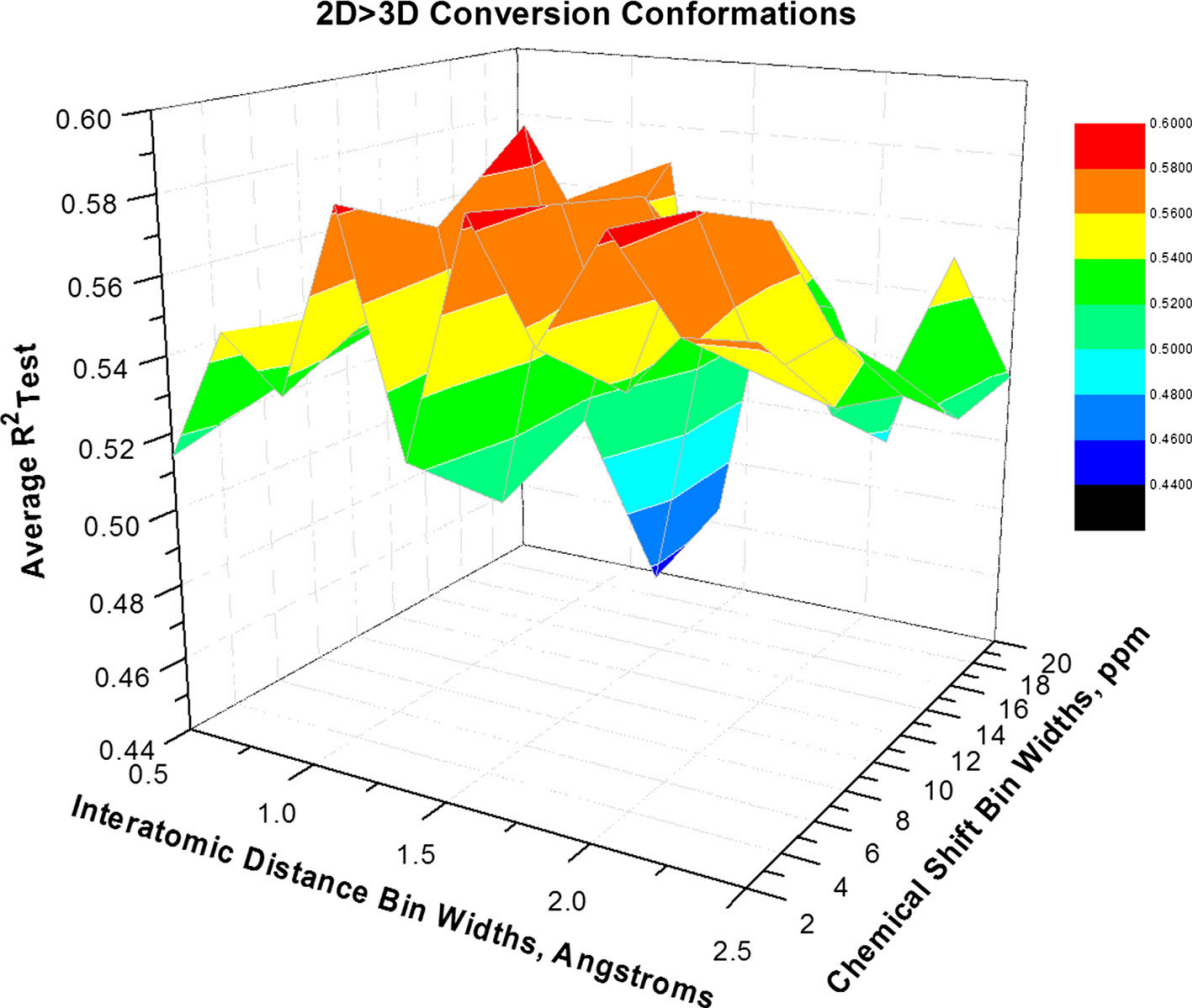
Average R_Test_^2^ response surface for direct 2D > 3D conversion modeled using 4 LVs. Conversion was executed using molecular mechanics via a Universal Force Field but involved no systematic energy optimization or alignment. Four optima are observed in the *red color* and they span the granularity range in both dimensions. In this figure the *color key* range is translated 0.02 R_Test_^2^ units higher than Fig. 2

We produced plots (Figs. 2, 3, 4) based on three of the conformation strategies and showing the average R_Test_^2^ response suface tessellated through relevant granularity combinations. In Fig. 2 several optima span the granularity range for Global Minimum Energy conformations.

Comparison of the Global Minimum Energy conformation response surface (Fig. 2) with a surface based on alignment (Fig. 3) showed significant differences. The overall response surface shape differed substantially, alignment being much simpler overall and significantly lowered on the right hand edge compared to Global Minimum Energy. The maximum R_Test_^2^ values were 0.58 or 0.56 and the lowest, 0.44 or 0.42, respectively, so the ranges from highest to lowest values were the same magnitude.

The results shown in Fig. 4 come from the study that used 3D conformations taken directly from an online source, ChemSpider. Since no systematic examination of conformations was required, the download process appeared instant. SDAR fingerprint composition, model building, and validation was completed 15 times faster than if systematic energy optimization and 30 times faster than if both optimization and alignment to a template were executed. The best 2D > 3D average R_Test_^2^ value was 0.61, for a composite model using 3LVs and 8 ppm × 8 ppm × 1.5 Å bins. This is 0.01 to 0.05 R_Test_^2^ units *higher* than the best composite models by other conformation strategies. For this experiment R_Scrambling_^2^ was only 0.05. Superior or even equivalent results for a model built without energy optimized or template adjusted conformations is a surprising result, one that is opposite to that found for other 3D descriptor types [28].

 Table 1 compares the analytical figures of merit for two Energy-Minimized composite models of different granularity; corresponding figures for two composite models based, respectively, on 50:50 or Best-of-Each-Compound 70:30 structural alignments; and results of 2D > 3D conversion. Comparing modified conformations, alignment gave *poorer* predictive quality than Global Minimum Energy optimized conformations (although according to a *t* test the difference was not statistically significant). This example suggests that, with respect to predictive accuracy, there would be no point in bothering to execute alignment strategies for 3D-SDAR modeling. The minimal utility of alignment for producing models with good statistical predictivity may be the appropriate conclusion for modeling interactions between substrates and a promiscuous receptor, a generally recognized characteristic of nuclear endocrine receptors, including the AR [29]. That inference might not hold for substrate interactions involving less promiscuous receptors. Also, the equivalence of models using no systematic conformational adjustments may hold only for data sets in which the strongly interacting molecules (here, steroids and their derivatives) are fairly rigid and their 3D conformations are invariant. The apparent superiority or equivalence of 2D > 3D predictions compared to that of the other methods tested was an unanticipated result [28].

**Table 1:** Model statistics as a function of experimental parameters

Conformation	Experimental parameters	*R*_Train._^2a^	*R*_Scrm._^2b^	R_Test_^2^	RMSD	Consensus R_Test_^2^			
						(a)	(b)	(c)	(d)
Global minimum energy (Model 1)	4 LV; 16 ppm; 1.0 Å	0.92	0.06	0.60	0.77	**0.62** +3.3 %			**0.65** +10 %
Global minimum energy (Model 2)	3 LV; 8 ppm; 1.0 Å	0.92	0.07	0.60	0.77		**0.58** −0.9 %	**0.64** +15 %	
Alignment, 50:50 electronic:steric	2 LV; 6 ppm; 1.0 Å	0.85	0.06	0.57	0.80				
Alignment, best-of-each	2 LV; 6 ppm; 1.0 Å	0.84	0.06	0.56	0.80				
2D > 3D conversion	3 LVs; 8 ppm; 1.5 Å	0.91	0.05	0.61	0.75				

Parameters were conformation basis, number of Latent Variables (LVs) in the PLS model, 3D-SDAR fingerprint granularity (chemical shifts in ppm; interatomic distances in Å) including predictive accuracy (R_Test_^2^) based on consensus predictions from composite models of differing granularity and conformation basis. (All R^2^ values in *non*-bold fonts are from composites based on averages from 100 random training/test set partitions)

^a^Replicate data for a similar estrogen receptor binding bioassay from the same data base, allowed calculation of an upper bound for modeling accuracy by the method of Doweyko et al. [30]. The calculation yielded R_Training_^2^ = 0.89 as the highest average that can be consistently obtained without over-fitting the data. All comparisons are based on R_Test_^2^, not R_Training_^2^ values. However, the best models in the R_Training_^2^ column are just slightly higher than 0.89, near the upper limit of the Doweyko criterion for the NCTR EDKB estrogen data. This could be a statistical artifact or could reflect somewhat greater accuracy for androgen compared to estrogen measurements

^b^All Y-axis R_Scrambling_^2^ values satisfy norms for modeling quality assurance. The low values indicate that these models could not be forced (for example, by the choice of the number of Latent Variables, number of descriptors or other modeling parameters) to fit randomized data

Table 1 also reports Consensus R_Test_^2^ results between (a) two Minimum Energy predictions of different granularity, (b) a Minimum Energy composite model and a 50:50 Alignment model of similar granularity, (c) a four component model (two Minimum Energy and two Alignment); and (d) a three component model, one each from Global Minimum Energy, Best-of-Each Alignment, and 2D > 3D Conversion (using MM via UFF with no alignment).

The (a) experiments averaging individual log(RBA) predictions yielded R_Test_^2^ = 0.62, an improvement of about 3.3 % relative to 0.60, the average of log(R_Test_^2^) from the two composite models. Improvement attributable to different granularities agreed with our earlier consensus results for other biological endpoints [1, 3, 4, 22].

In (b), consensus predictions of models with similar granularity but different molecular conformations yielded R_Test_^2^ = 0.58, a *decrease* of 0.9 % relative to the average of corresponding statistics (0.585). In this case, minimum energy and alignment conformations did not extract different structure–activity information from SDAR descriptors.

In (c), consensus of four composite models, yielded an R_Test_^2^ = 0.64, an improvement of 15 % relative to the average, 0.5825, of corresponding statistics.

In (d), a consensus from three composite models, one of which was the best model using 2D > 3D ChemSpider conformations (R_Test_^2^ = 0.61), gave R_Test_^2^ = 0.65, an improvement of 10.0 % relative to the average R_Test_^2^ values of the three composite models.

Improved statistical results by consensus may justify modeling with conformations defined in more than one way. The question remains whether it is worth the extra time and effort. This question is compounded by the fact that it is possible to interpret less than optimal SDAR models, even ones built using the expedited 2D > 3D process. If the ability to infer association and discover toxicophores is deemed more important than incremental improvements in predictive accuracy, then increasing R_Test_^2^, even by up to 15 %, may be unnecessary.

**Fig. 5:**
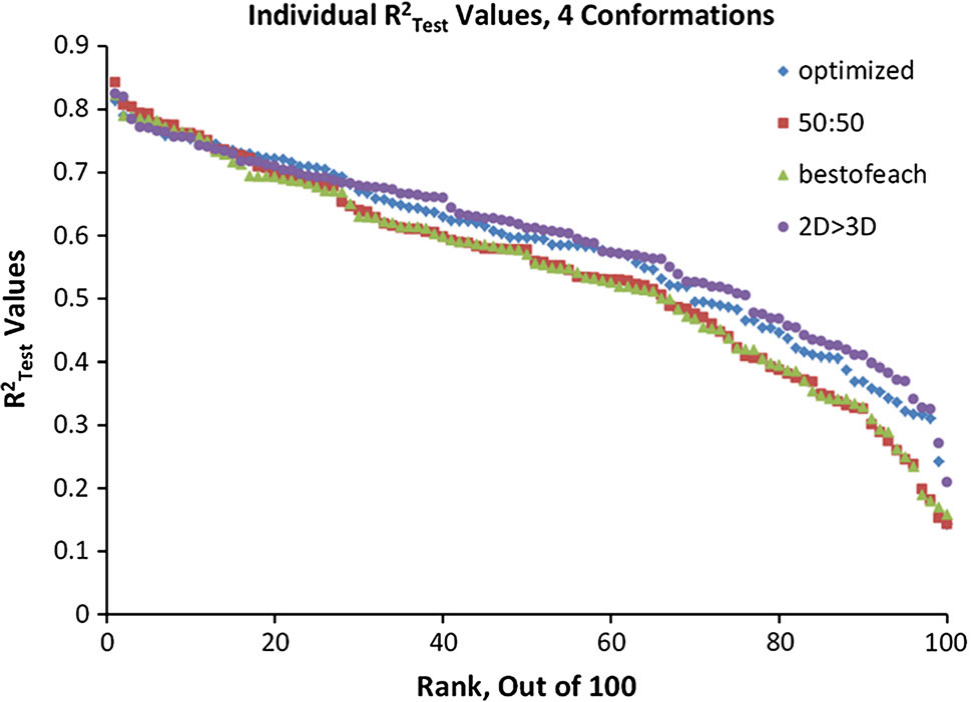
Overlaid plots of ranked R_Test_^2^ values. These are based on the optimum granularity (bin dimensions giving the highest average R_Test_^2^) of each conformational variant. The four variants used symbols *blue diamond*, *red square*, *green triangle*, or *purple circle*, respectively, for conformations based on Global Minimum Energy (4 LV, 16 ppm, 1 Å), Alignment 50:50 (2 LV, 6 ppm, 1.0 Å), Best-of-Each Alignment 70:30 (2 LV, 6 ppm, 1.0 Å), or 2D > 3D conversion (3 LV, 8 ppm, 0.5 Å). For each series, test results varied greatly as a function of the partition between training and test sets, which shows why only average or median values would provide objective comparison among experiments

 Figure 5 is a plot of R_Test_^2^ values for the 100 training/test set partitions in the best models of each conformation mode, separately ranked from highest to lowest. The number of LVs used in optimal models plotted varied from 2 to 4. In each case, the range of R_Test_^2^ values is quite broad, typically between 0.85 and 0.20. Merely by selecting different training and test set partitions, it is possible to generate models varying in predictivity from extraordinarily good to unacceptably poor. The plot shows similarity in the optimal values predicted, albeit using different partitions. The two experiments using aligned conformations were more sensitive to unfavorable data partitions as shown by their lower trajectory on the right hand side of the plots. This depressed trajectory shows why the average R_Test_^2^ values by alignment were lower than those of the other two conformation strategies. A likely explanation is that when molecules are aligned, any mistakes in alignment show up as reduced predictive accuracy whenever most of the misaligned molecules appear in the hold out test set.

The only objective way to make fair comparison of results is to partition by an agnostic method (e.g., an RNG) and report some measure of the resulting distributions’ central tendencies—averages or means, derived from an identical sequence of partitions. We used 100 RNG partitions here because our studies have shown that using fewer than 100 yielded an inflated average R_Test_^2^, up to 10 % higher, whereas averages from more than 100 yielded approximately the same metrics and only increased computational overhead [4]. When the RNG-generated 100-partition protocol was followed, and predictions for each compound were averages calculated from hold-out test set predictions, the resulting regression of average predictions against experimental values closely approximated the corresponding predictive accuracy for a completely external test set [22]. Agreement between hold-out test set and external test set predictive accuracy is the appropriate goal because it shows that the value of the model for predicting unknown compounds has been accuratetly assessed.

**Fig. 6:**
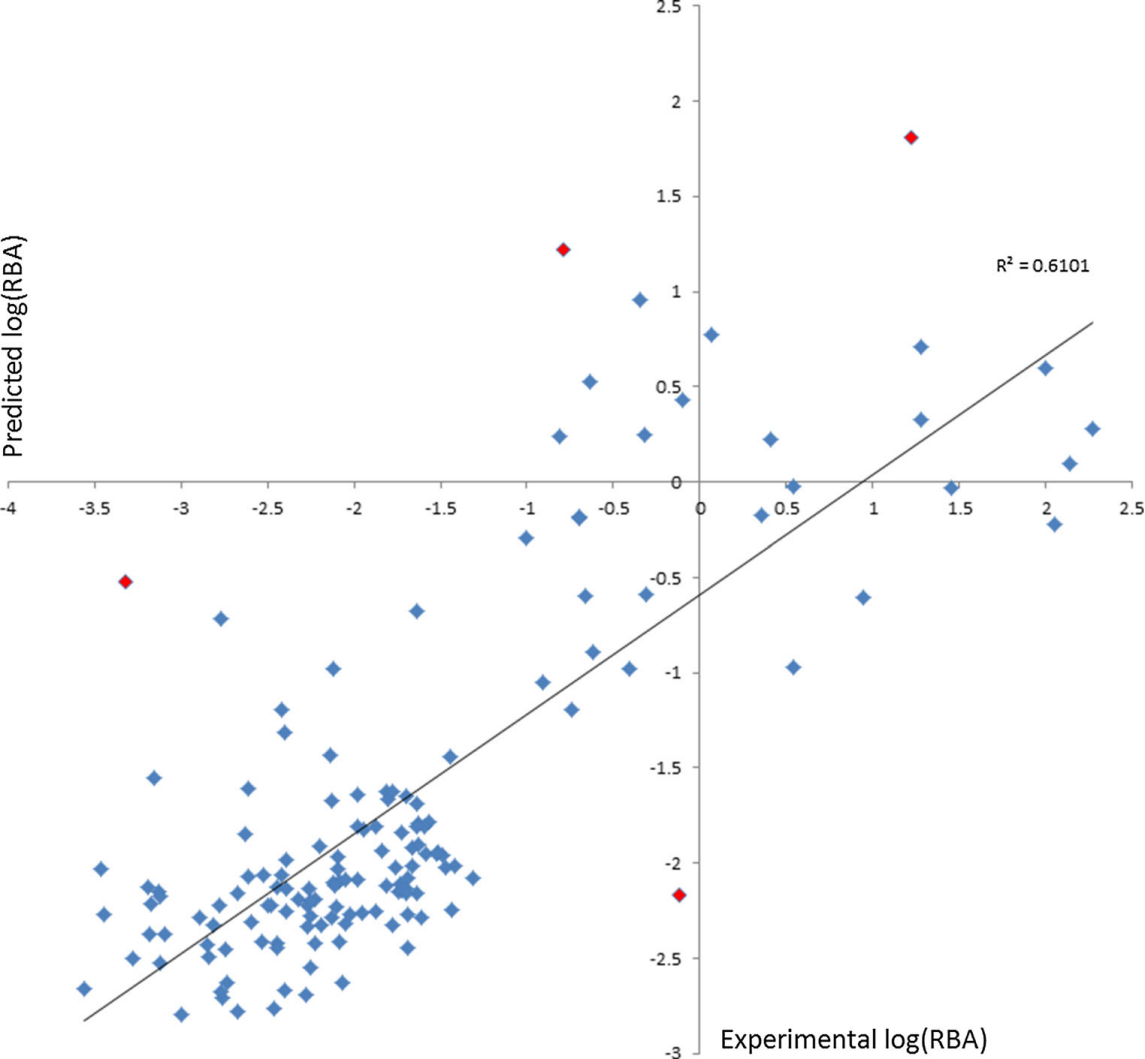
Hold-out test set predicted versus experimental log(RBA) plot. Data are for the 2D > 3D direct conversion model with granularity 8 ppm × 1.5 Å derived from 3LVs. Note that the spread of predictions is similar across the range of values, particularly that the upper log(RBA) range is not more accurately predicted than the lower even though a higher proportion of the more active structures are inflexible. In these models, most major excursions from the regression line overestimated androgenicity, an error tendency useful for conservative toxicity screening. See “Discussion” section for comments on the four data points indicated by *red diamonds*

As reported in Table 1, the best composite based only on direct 2D > 3D Conversion yielded R_Test_^2^ = 0.61: e.g., it was the most accurate of the composite models, an interesting and unanticipated result. Figure 6 plots the predicted versus experimental log (RBA) values for this 2D > 3D conversion model. The bins had granularity 8 ppm × 1.5 Å.

### Structure alert discovery from 2D > 3D models

The direct download models might not prove as useful for discovering structure alerts, particularly in the interatomic distance dimension, since some of the compounds will have been modeled in local optimum conformations thus presumably decreasing predictive accuracy for their structures (though decreased predictive accuracy was not observed in the 2D > 3D case modeled here).

The possibility of structure alert dicovery was explored by extracting important bins from the best 2D > 3D model and overlaying them on the strongest androgen receptor binder, dihydrotestosterone, an inflexible molecule (Kier Index, 2.14). This way of working backward from important bins to structural motifs has proved enlightening when the conformations modeled were Global Minimum Energy. The process of identifying and mapping important bins from a model can be executed no matter the conformational mode, including 2D > 3D. The relevant issue is whether identified toxicity-associated substructures are consistent with those determined in a systematic way and also make sense in explaining, in this case, a molecule’s AR binding.

**Fig. 7:**
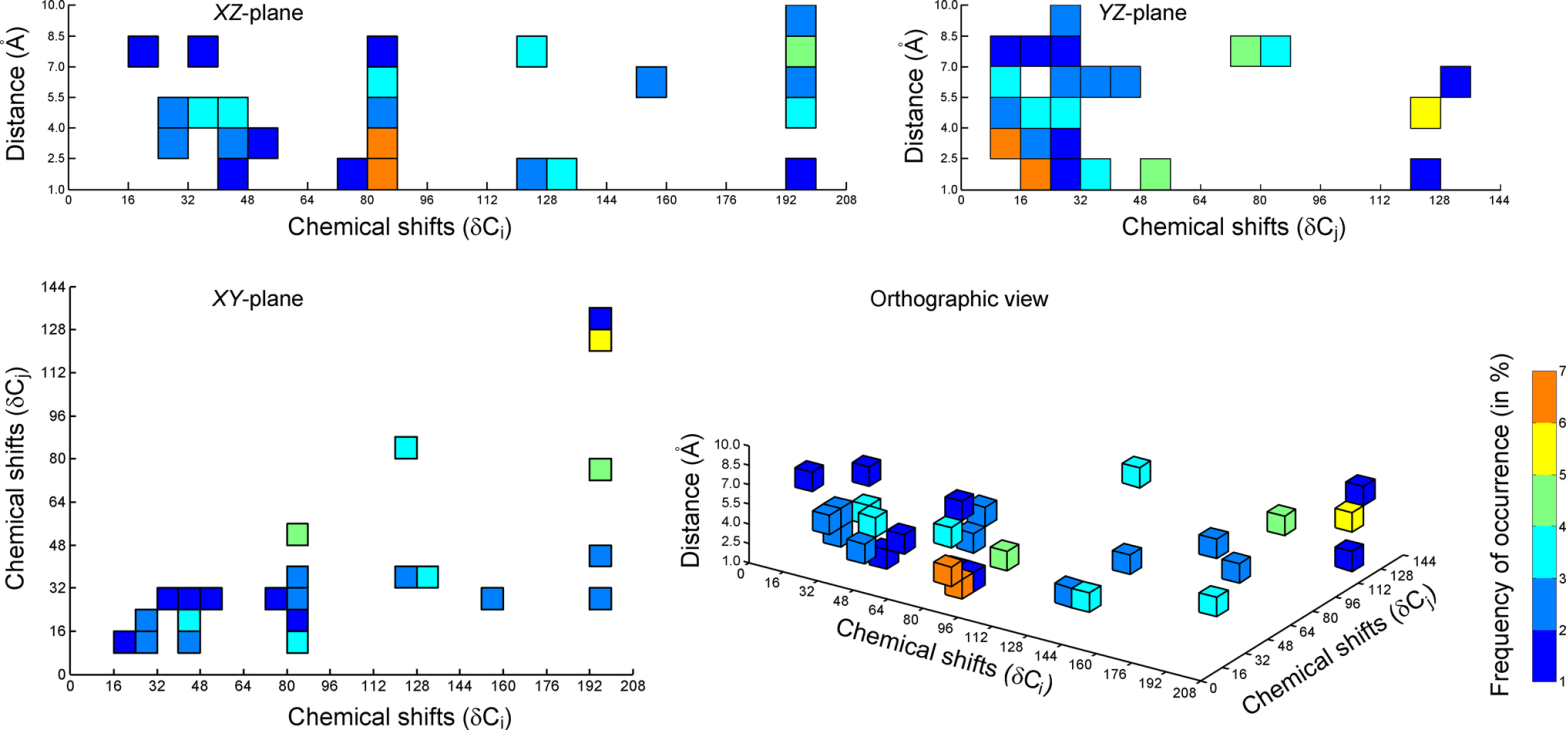
Plot of 3D SDAR fingerprint space. This plot shows important bins associated with AR binding from a 2D > 3D composite model with bin width dimensions 8 ppm × 8 ppm × 1.5 Å based on 3 LVs giving average R_Test_^2^ = 0.61. A bin is “important” only if it is both highly weighted and frequently occurring as highly weighted within the 100 randomized models

Color coded to reflect the final ranking based on percent occupancy, a few important bins from this model are shown on the 3D map of QSDAR space (Fig. 7).

**Fig. 8:**
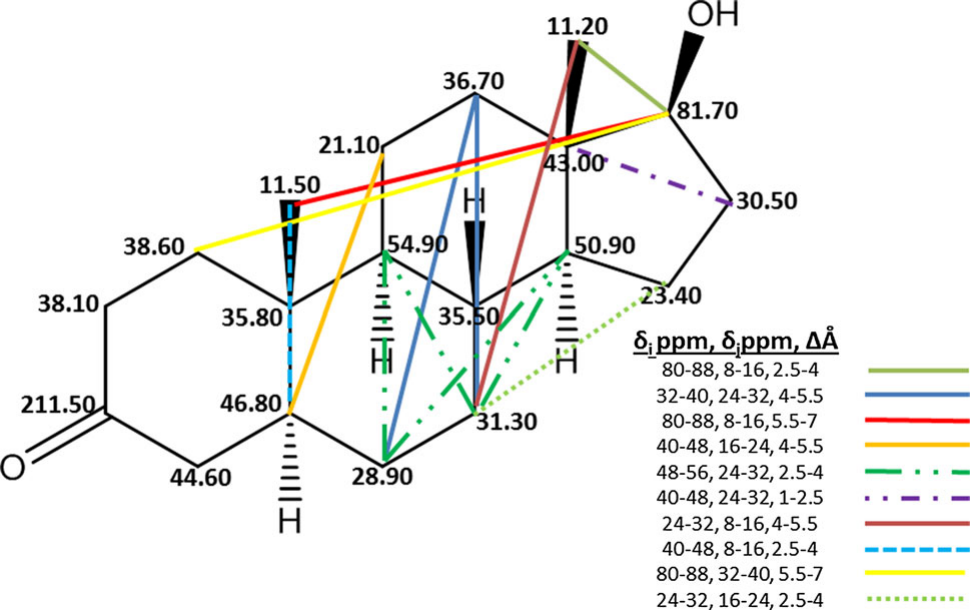
2D structure of dihydrotestosterone (Compound-82). It is annotated with ^13^C chemical shifts and overlaid with colored lines, each indicating an important bin discovered from the 2D > 3D composite model based on 3 LVs and having granularity 8 ppm × 1.5 Å and average R_Test_^2^ = 0.61. In dihydrotestosterone, for 10 important bins, one occurs in four instances and another in two. Thus the estimated log(RBA) would be the sum of 14 weighted contributions (plus a few more not shown from lower or negatively weighted bins)

Important bins were used to discover toxicophores. Select important bins were mapped onto dihydrotestosterone, the most strongly binding molecule, as shown in Fig. 8. To facilitate the examination, we ranked the bins by percent occupancy then, starting from the top, identified every molecule in the training/test set in which that bin was occupied. For Fig. 8, whenever dihydrotestosterone appeared in the list for an important bin, that bin’s identity was marked on the 2D dihydrotestosterone structure. Such bins were represented as colored dotted or dashed lines. The lines visualize many of the AR binding structure–activity relationship components.

Via the model, any molecule’s estimated binding affinity is calculated as the sum of products of its occupied bins multiplied by their respective weights. It is not surprising that strongest binding to AR occurs for a molecule, dihydrotestosterone, with 10 highly weighted and frequently occupied (important) bins and a number of lesser bins contributing to the estimated log(RBA) total. This fact demonstrates the ability to deduce structural associations of androgeneicity from 2D > 3D SDAR models.

### Investigation of 2D > 3D modeling for endpoints other than AR binding

If acceptable predictive accuracy were observed for modeling other endpoints, the 3D-QSDAR technique should be practically adaptable for rapidly modeling large data sets. We tested two other endpoints previously modeled using Global Minimum Energy conformations to see whether results by 2D > 3D conformations were comparable in predictive accuracy. For estrogen receptor (ER) binding, the best 2D > 3D model gave average R_Test_^2^ = 0.55. This compares to average R_Test_^2^ = 0.56 for our best log(ER) binding model where Global Minimum Energy was used for the conformations [1]. The ER binding receptor and data set were similar to the AR binding receptor and data set modeled here. The Kier flexibility of the 130 estrogens comprised 41 rigid (31.5 %), 58 partially flexible (44.6 %), and 31 flexible (23.8 %) structures. The corresponding percentages for the androgens were 32.9, 47.9, and 19.2 %, respectively. Thus, as with the androgens, use of directly downloaded 3D *.mol files worked as well as energy minimized ones for the estrogens. This is consistent with the strong binder rigidity hypothesis advanced in the preceding paragraph.

For acute toxicity, modeling 154 diverse structures, the corresponding compound numbers and flexibility ranges were 41 (26.6 %), 69 (44.8 %), and 44 (28.6 %). That is, compared to the androgens and the estrogens, for the acute toxicity data set the percentage of flexible structures was higher and partially flexible or inflexible structures, lower. The best 2D > 3D acute toxicity model yielded an average R_Test_^2^ = 0.63. This compares to average R_Test_^2^ = 0.77 for the best PLS composite model of the same endpoint when Global Minimum Energy conformations were used [4]. This shows that in some cases 3D-SDAR is sensitive to conformation definition. This result is also consistent with the hypothesis that greater modeling vulnerability to conformation is observed when structures are flexible, when the most rigid compounds are not necessarily the most toxic, and/or interaction with more than one target affects the toxic endpoint.

When using the 3D QSDAR method it appears that 2D > 3D conformations may work as well as or better than other methods for generating conformations when modeling endocrine system nuclear receptors such as AR and ER, but more poorly for mechanistically ambiguous endpoints like acute toxicity.

## Discussion

Examination of Fig. 8 shows that molecular features associated with a steroid backbone are important for dihydrotestosterone’s strong binding to AR. This is not surprising since the majority of strong AR binders are steroids. Three of the bins involve the same atom, the one having a chemical shift (81.70 ppm) most affected by the presence of an adjacent hydroxyl substituent. Having such a chemical shift is not a universal steroid characteristic. Similarly, aromaticity in the steroid A ring, a common feature for steroidal estrogens, is *not* associated with a strong androgen: for dihydrotestosterone the three important bins with a carbon in that ring include ^13^C chemical shifts associated with *sp*^3^ hybridization. It is significant that, though these bins were discovered from a model based on less coherent conformations, all but two of the important bins shown in Fig. 8 involve longer distance atom pair relationships that for flexible compounds can vary with 3D molecular conformation. In summary, structure alerts discovered from the 3D-QSDAR composite model reflect contributions from the molecular biology influenced by the most frequent structural scaffold used in model construction if that scaffold is also associated with strong AR binding.

There was not an improvement in predictive accuracy for models built on aligned conformations compared to lowest energy conformations. Rather, the predictive accuracy of structurally aligned conformations, whether forced to template using default parameters or using parameters selected as optimal for their template, was measurably poorer than that of any alternative conformational mode tested. The 2D > 3D model using neither global lowest energy nor alignment gave improved predictivity. It appears that 3D-SDAR, although its compounds are represented in 3D, is relatively insensitive to alignment and energy optimization for modeling AR, ER, or similar receptors modulating critical reproductive functions. A significant benefit is that models built on 2D > 3D conversion take a small fraction of the model construction work compared to energy-minimization or alignment-based models, only 7 or 3 %, respectively.

It should not be a surprise that many natural substrates involved in critical signaling pathways are built on fairly rigid carbon scaffolds (*i.e*., fused ring systems, aromatic rings). These are presumably less vulnerable to catastrophic inactivation via enzyme/receptor mutation and more optimal than highly flexible molecules for assuring proper fit (“lock and key” or “molded fit”) between the substrate and the corresponding active site [31]. Thus, it is possible that a significant portion of processes critical for reproduction can be successfully modeled using 2D > 3D conformations.

The ability to create even an inferior but possibly acceptable model (*e.g*., average R_Test_^2^ = 0.55 for acute toxicity) using 2D > 3D conformations in 3–7 % of the time and apply it for prediction of a very large number of compounds by merely downloading their 3D *.mol files would facilitate rapid screening of vast chemical libraries whenever the training/hold-out-test set results warrant such an extension.

The R_Test_^2^ response surfaces differ among energy-optimized, template-aligned, and 2D > 3D conformations. The response surfaces for the two types of template-aligned conformations were similar to each other and quite different from each of the other two. Significant differences in the response surface contours suggest that the androgen data set included enough flexible or partly flexible molecules to assess the question of conformational mode dependency in 3D-QSDAR.

From the best 2D > 3D model we chose as an experiment to omit four outliers, 16β-hydroxy-16-methyl-3-methyl-estradiol (Compound 53), testosterone propionate (Compound 137), *l*-norgestrel (Compound 118), and 2,4,5-trichlorophenoxyacetic) acid (Compound 6). This improved average R_Test_^2^ from 0.61 to 0.67, or 10 %. In the end, we did not exclude outliers in order to improve statistics, but have reported this result to demonstrate how sensitive overall statistical predictivity is to the exclusion of a few poor-quality predictions and how misleading results can be if compounds are arbitrarily excluded, especially without disclosure and without structural or at least statistical justification. For objectivity, having selected a test set, the modeler should not exclude problem compounds merely to inflate results.

RNG-generated data set partitions provided an objective but conservative training/test selection basis and 20 % hold-out, a rigorous validation standard. Thus, average R_Test_^2^ results from 100 partitions might have suffered when naively compared to statistical figures of merit for models not so rigorously validated. Such factors considered, results in Table 1 compare favorably to other published AR QSAR models. Loughney and Schwender, modeling 48 androgens, achieved Leave-One-Out cross validation (LOO) Q^2^ = 0.525 [32]. Modeling by CoMFA the same 146 compounds studied here and using the same experimental AR binding data, Hong et al. obtained LOO Q^2^ = 0.571 [33]. In both cases, 3D-QSDAR produced more accurate predictions under much more rigorous validation.

Recognized characteristics of CoMFA modeling are its requirements for identification of bioactive conformers as well as accurate alignment of ligands to each other, and that these pose particular challenges with large scale modeling projects estimating activity for huge compound numbers [34]. The work presented here shows that 3D-QSDAR, under some circumstances, can model as well as or better than CoMFA without conformational adjustment or receptor site alignment. The tedious nature of alignment should be obvious from the experimental descriptions in the subsection above dealing with conformational comparison experimental design. Avoiding this necessity while still executing effective modeling represents a significant technical advance and commends 3D-SDAR for large data set modeling and screening.

Many *n*D-QSAR methods (e.g., Eigen Value Analysis) cannot extract useful information for drug design and toxicophore identification from PLS models [10]. This work has demonstrated that information extraction related to biological affinity is possible from 3D-SDAR PLS models and that toxicity-structure associations can be derived from such models.

## Conclusions

For modeling interactions with AR, we observed no improvement but rather deterioration in model predictive accuracy associated with Template-Aligned conformations compared to Global Minimum Energy conformations. On the contrary, improved predictivity was obtained using downloaded 3D conformations without systematic molecular mechanics adjustment. Downloaded conformations were acquired almost instantly and their use bypassed the most time consuming and, for alignment, semi-subjective portions of the modeling process. The different conformation strategies reached optimal performance under different modeling parameters. Improved prediction accuracy was typically obtained from consensus of models based on different conformations, because different conformations produced different outliers. Consensus improvements were significant and might justify the extra effort required to perform alignment. 3D-SDAR modeling identified structural alerts of androgen receptor affinity by mapping important 3D-SDAR bins onto the chemical structures of compounds with high affinity. This could have been done using models built on any of the conformation bases but was exemplified for 2D > 3D.

We hypothesized which substrate and receptor characteristics would allow for rapid and accurate modeling using only 2D > 3D mol files. 2D > 3D direct download can be implimented by testing the quality of models via a rigorous 20 % Hold Out and 100 random generated training/test set partitions. If hold-out test set results are acceptable, there is good likelihood that affinity predictions for unknown compounds using their 2D > 3D conformations will also be as accurate.

## Electronic supplementary material

Below is the link to the electronic supplementary material.

Supplementary material 1 (PDF 96 kb)

Supplementary material 2 (PDF 863 kb)

Supplementary material 3 (XLSX 44 kb)

Supplementary material 4 (XLSX 60 kb)

Supplementary material 5 (XLSX 52 kb)

Supplementary material 6 (XLSX 52 kb)

Supplementary material 7 (XLSX 53 kb)

Supplementary material 8 (PDF 101 kb)

Supplementary material 9 (XLS 46 kb)
