# Supplementary material for: Alignment-independent technique for 3D QSAR analysis
Source: J Comput Aided Mol Des. 2016 Mar 30;30:331–45. doi: 10.1007/s10822-016-9909-0 (PMC4833814; doi:10.1007/s10822-016-9909-0)
Supplement: Supplementary file 1 — Supplementary material 1 (PDF 96 kb) [file 10822_2016_9909_MOESM1_ESM.pdf]

For *Journal of Computer-Assisted Molecular Design* Online Resource_1

Alignment Independent Technique for 3D QSAR Analysis

Jon G. Wilkes*[1], Iva B. Stoyanova-Slavova[1], Dan A. Buzatu[1]

1 Division of Systems Biology at the National Center for Toxicological Research, 3900 NCTR Road, Jefferson, AR 72079 USA

*Corresponding Author:
Jon G. Wilkes
NCTR
3900 NCTR Road
Jefferson, AR 72079
T: 1 + (870)543-7108
F: 1+ (870)543-7086
E: jon.wilkes@fda.hhs.gov

ESM_1. Androgenicity Background and Regulatory Responsibilities

The androgen receptor, natural and synthetic androgens

Nuclear receptors, in this case the androgen receptor (AR), mediate effects of hormones and other endogenous ligands to control development and metabolism [11]. The AR is a member of the nuclear receptor superfamily and is one of five vertebrate steroid receptors [12]. It is required for the growth and maintenance of the prostate [13]. An example of AR-modulated male disease is prostate cancer, cells of which depend primarily on AR for growth and survival [14]. Prostate cancer is quite common and the second leading cause of cancer deaths in men in the United States [15].

AR function is regulated by the binding of androgens, which initiate sequential conformational changes in the receptor that affect receptor-protein and receptor-DNA interactions [12]. Androgens must have specific characteristics, including an appropriate size and conformation, in order to bind to the AR. For endogenous steroid hormones, the molecular structure is rigid, so that an appropriate conformation is assured. The strongest-binding endogenous androgens are testosterone and dihydrotestosterone, both steroids. Synthetic chemicals, natural products, or medicines (*e.g.*, tamoxifen) may have flexible molecular structures for which some conformations, but not all, will fit into and interact with the AR binding site.

If released in quantity into the environment *(e.g.*, into air, water, food, feed, *etc.*), synthetics can cause deleterious effects in animal life by interfering with the endocrine system, including the AR. They modulate endocrine activity by mimicking or blocking the actions of endogenous hormones [16]. Numerous chemicals have been found that cause endocrine disrupting effects in experimental laboratory animals. Exposures are also correlated to such effects in wildlife [17].

Regulatory responsibilities and the need for *in silico* predictions

The Food and Drug Administration and other regulatory authorities monitor many compounds with known or suspected endocrine activity. More troubling are the approximately 100,000 chemicals in commercial production, for only about 600 of which workplace air-quality standards have been set.[1] The rest have not been studied sufficiently to regulate by the US Occupational Safety and Health Administration (OSHA), the FDA, the EPA, *etc.* These compounds and their environmental degradation products and metabolites comprise a number far too great to test in animals or even to assay by *in vitro* methods. The number of such chemicals is increasing. Consequently, there is a need to develop efficient and economical *in silico* screening procedures for endocrine disrupting chemicals.

References

11. Luccio-Camelo DC, Prins GS. Disruption of androgen receptor signaling in males by environmental chemicals. J Steroid Biochem Mol Biol. 2011; 127: 74-82.

12. Gao W, Bohl CE, Dalton JT. Chemistry and structural biology of androgen receptor. Chem Rev. 2005; 105: 3352-3370.

13. Basu S, Tindall DJ. Androgen action in prostate cancer. Horm Cancer. 2010; 1: 223-228.

14. Marques RB, Dits NF, Erkens-Schulze S, van IJcken WFJ, van Weerden WM, Jenster G. Modulation of Androgen Receptor Signaling in Hormonal Therapy-Resistant Prostate Cancer Cell Lines. PLoS One. 2011; 6(8): e23144.

15. Feldman BJ, Feldman D The development of androgen-independent prostate cancer. Nat Rev Cancer. 2001; 1: 34-45.

16. Lill MA, Vedani A. Computational modeling of receptor-mediated toxicity. In Elkins S, editor. Computational toxicology: risk assessment for pharmaceutical and environmental chemicals, Hoboken, New Jersey: John Wiley and Sons, Inc.: 2007. pp 315-351.

[1] http://ecb.jrc.it/esis/; https://www.osha.gov/dsg/annotated-pels/tablez-1.html; https://www.osha.gov/dsg/annotated-pels/tablez-2.html; https://www.osha.gov/dsg/annotated-pels/tablez-3.html

17. Colborn T, vom Saal FS, Soto AM Developmental effects of endocrine-disrupting chemicals in wildlife and humans. Environ Health Perspect. 1993; 101: 378-384.
