# Supplementary material for: Alignment-independent technique for 3D QSAR analysis
Source: J Comput Aided Mol Des. 2016 Mar 30;30:331–45. doi: 10.1007/s10822-016-9909-0 (PMC4833814; doi:10.1007/s10822-016-9909-0)
Supplement: Supplementary file 2 — Supplementary material 2 (PDF 863 kb) [file 10822_2016_9909_MOESM2_ESM.pdf]

For *Journal of Computer-Assisted Molecular Design* Online Resource_2

Alignment Independent Technique for 3D QSAR Analysis

Jon G. Wilkes*[1], Iva B. Stoyanova-Slavova[1], Dan A. Buzatu[1]

1 Division of Systems Biology at the National Center for Toxicological Research, 3900 NCTR Road, Jefferson, AR 72079 USA

*Corresponding Author:
Jon G. Wilkes
NCTR
3900 NCTR Road
Jefferson, AR 72079
T: 1 + (870)543-7108
F: 1+ (870)543-7086
E: jon.wilkes@fda.hhs.gov

ESM_2. The 146 androgens, their template alignment compounds, experimental AR log(RBA) values, and 2D structures

#	Name (Template - #)	Log(RBA)	Chemical Identity		
			**Class**	**CAS#**	**2D Structure**
1	1-methoxy-4-[1-propenyl]-benzene (4-hydroxybiphenyl-34)	-3.19	other	4180-23-8	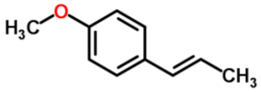
2	2-(4-hydroxy-benzyl)-isoindole-1,3-dione (2-(4-nitrobenzyl)-1H-isoindole-1,3(2H)-dione-3)	-2.76	indole	24124-24-1	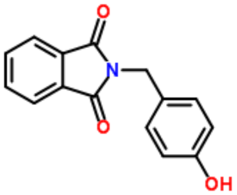
3	2-(4-nitrobenzyl)-1H-isoindole-1,3(2H)-dione (2-(4-nitrobenzyl)-1H-isoindole-1,3(2H)-dione-3)	-2.46	indole	62133-07-7	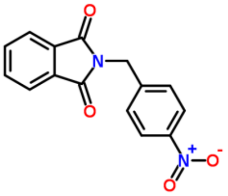
4	2,2',4,4'-tetrachlorobiphenyl (4-hydroxybiphenyl-34)	-1.74	PCB	2437-79-8	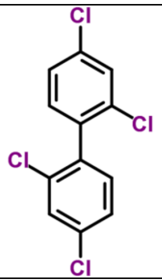
5	2,3,4,5-tetrachloro-4'-biphenylol (4-hydroxybiphenyl-34)	-1.73	PCB	67651-34-7	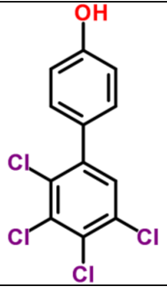

6	(2,4,5-trichlorophenoxyacetic) acid (6-hydroxyflavanone-51)	-3.18	pesticide	93-76-5	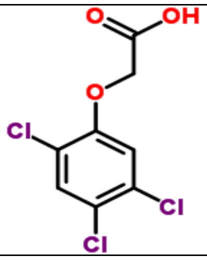
7	2,4'-dichlorobiphenyl (4-hydroxybiphenyl-34)	-1.72	PCB	34883-43-7	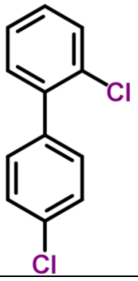
8	2,4-dihydroxybenzophenone (dihydroxymethoxychlor olefin-83)	-2.53	DDT	131-56-6	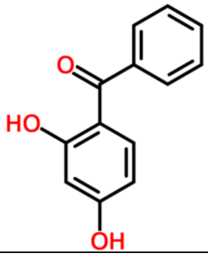
9	2-benzyl-isoindole-1,3 dione (2-(4-nitrobenzyl)-1H-isoindole-1,3(2H)-dione-3)	-3.12	indole	2142-01-0	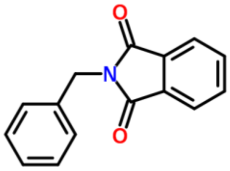
10	2-hydroxyestradiol (dihydrotestosterone-82)	-1.44	steroid	362-05-0	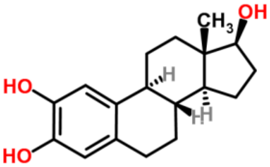
11	2-sec-butylphenol (*p*-nonylphenol-114)	-2.52	phenol	89-72-5	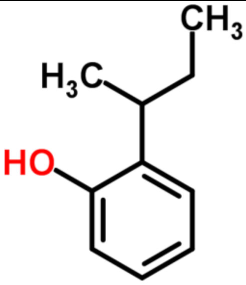

12	3,3',5,5'-tetrachloro-4,4'-biphenyldiol (4-hydroxybiphenyl-34)	-2.10	PCB	13049-13-3	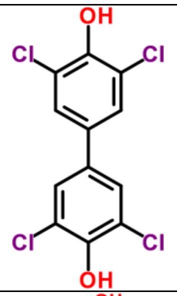
13	3,3'-dihydroxyhexestrol (4-hydroxy-tamoxifen-40)	-2.08	DES	79199-51-2	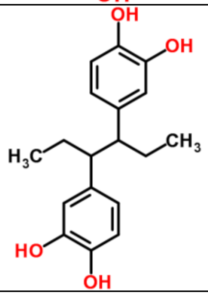
14	3,4-diphenyltetrahydrofuran (6-hydroxyflavanone-51)	-1.98	other	93433-53-5	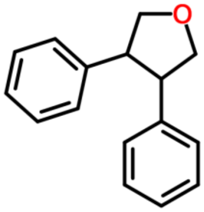
15	3α-androstanediol (dihydrotestosterone-82)	-0.81	steroid	1852-53-5	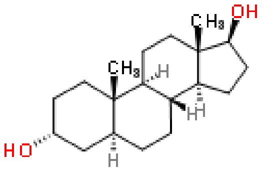
16	3β-androstanediol (dihydrotestosterone-82)	0.36	steroid	571-20-0	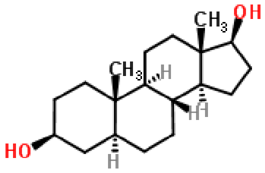

17	3-chlorophenol (*p*-nonylphenol-114)	-3.17	phenol	108-43-0	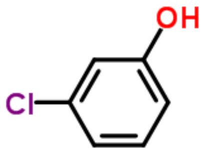
18	3-deoxyestradiol (dihydrotestosterone-82)	0.54	steroid	2529-64-8	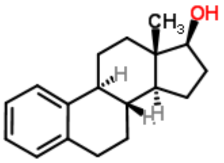
19	3-methyl estriol (dihydrotestosterone-82)	-2.25	steroid	3434-79-5	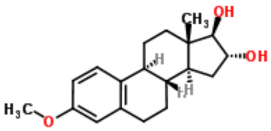
20	4-(3,5-diphenylcyclohexyl)phenol (*p*-nonylphenol-114)	-2.27	phenol	33330-65-3	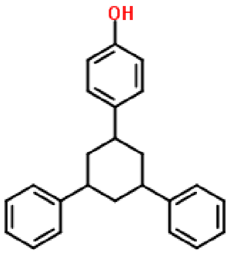
21	4-(benzyloxyl)phenol (*p*-nonylphenol-114)	-2.89	phenol	103-16-2	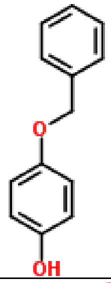
22	4,4'-dihydroxy benzophenone (dihydroxymethoxychlor olefin-83)	-2.67	DDT	611-99-4	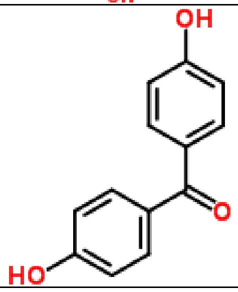

23	4,4'-dihydroxystilbene (4-hydroxy-tamoxifen-40)	-2.44	DES	659-22-3	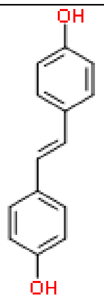
24	4,4'-sulfonyldiphenol (dihydroxymethoxychlor olefin-83)	-3.09	DDT	80-09-1	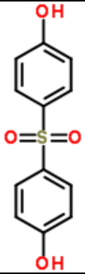
25	4-amino butylbenzoate (di-*n*-butyl phthalate-88)	-2.85	other	94-25-7	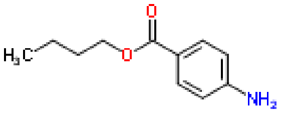
26	4-androstenediol (dihydrotestosterone-82)	-0.31	steroid	1156-92-9	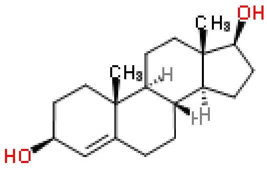
27	4-androstenedione (dihydrotestosterone-82)	-0.62	steroid	63-05-8	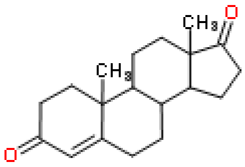

28	4-chloro-2-methyl phenol (*p*-nonylphenol-114)	-2.59	phenol	1570-64-5	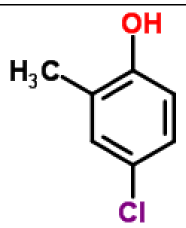
29	4'-chloroacetoacetanilide (2-(4-nitrobenzyl)-1H-isoindole-1,3(2H)-dione-3)	-3.46	flutamide	101-92-8	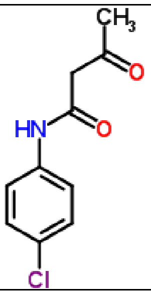
30	4-dodecylphenol (*p*-nonylphenol-114)	-1.81	phenol	104-43-8	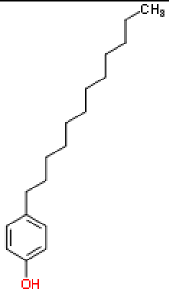
31	4-heptyloxybenzoic acid (α-zearalenol-146)	-2.74	other	15872-42-1	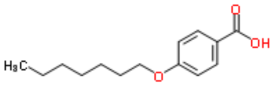
32	4-heptyloxyphenol (*p*-nonylphenol-114)	-1.69	phenol	13037-86-0	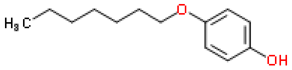

33	4-hydroxybenzophenone (dihydroxymethoxychlor olefin-83)	-2.78	DDT	1137-42-4	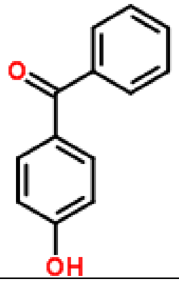
34	4-hydroxybiphenyl (4-hydroxybiphenyl-34)	-1.43	PCB	92-69-3	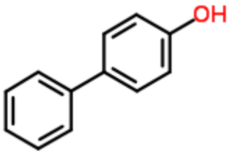
35	4'-hydroxychalcone (6-hydroxyflavanone-51)	-2.27	phyto	2657-25-2	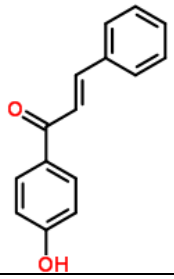
36	4-hydroxychalcone (6-hydroxyflavanone-51)	-2.19	phyto	20426-12-4	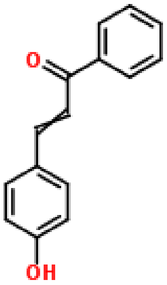
37	4'-hydroxyflavanone (6-hydroxyflavanone-51)	-2.48	phyto	6515-37-3	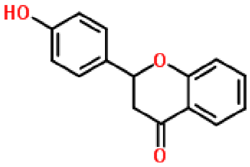

38	4-n-octylphenol (*p*-nonylphenol-114)	-1.81	phenol	1806-26-4	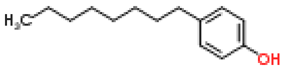
39	4-hydroxy-estradiol (dihydrotestosterone-82)	-0.91	steroid	5976-61-4	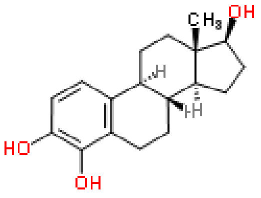
40	4-hydroxy-tamoxifen (4-hydroxy-tamoxifen-40)	-1.49	DES	68047-06-3	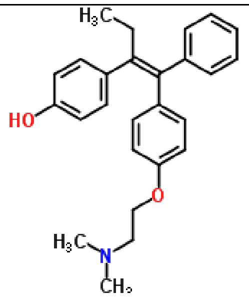
41	4-sec-butylphenol (4-hydroxy-tamoxifen-40)	-2.44	phenol	99-71-8	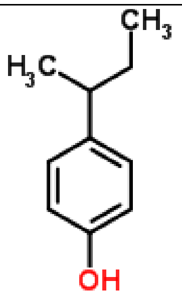
42	4-tert-amylphenol (*p*-nonylphenol-114)	-2.39	phenol	80-46-6	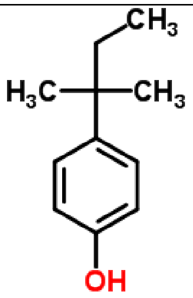

43	4-tert-butylphenol (*p*-nonylphenol-114)	-2.67	phenol	98-54-4	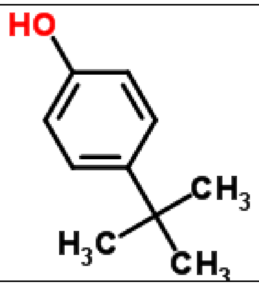
44	5,6-didehydroisoandrosterone (dihydrotestosterone-82)	-1.98	steroid	53-43-0	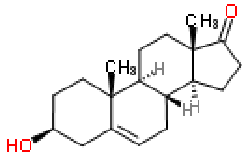
45	5α-androstan-3β-ol (dihydrotestosterone-82)	-0.74	steroid	1224-92-6	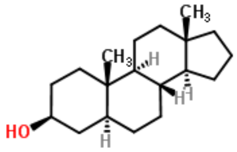
46	5α -androstan-17β–ol (dihydrotestosterone-82)	1.45	steroid	1225-43-0	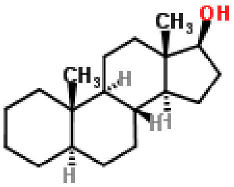
47	5α-androstane (dihydrotestosterone-82)	-3.32	steroid	438-22-2	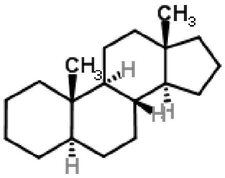

48	5α-androstane-3,11,17-trione (dihydrotestosterone-82)	-1.64	steroid	1482-70-8	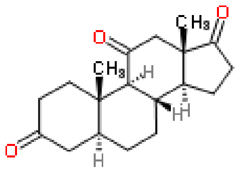
49	6α-methyl-17α-acetoxyprogesterone (dihydrotestosterone-82)	0.94	steroid	71-58-9	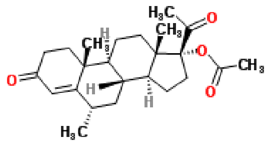
50	6α-methyl-17α-hydroxy-progesterone (dihydrotestosterone-82)	-0.41	steroid	520-85-4	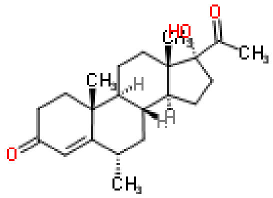
51	6-hydroxyflavanone (6-hydroxyflavanone-51)	-1.78	phyto	4250-77-5	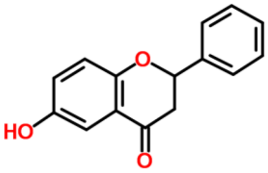
52	6-hydroxyflavone (6-hydroxyflavanone-51)	-2.77	phyto	6665-83-4	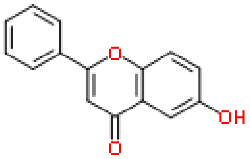

53	16β-hydroxy-16-methyl-3-methyl-estradiol (dihydrotestosterone-82)	-2.08	steroid	5108-94-1	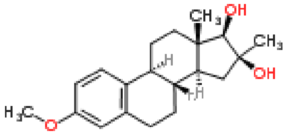
54	17α-estradiol (dihydrotestosterone-82)	-2.40	steroid	57-91-0	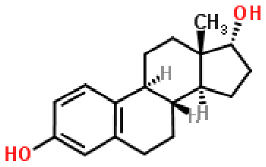
55	17β-estradiol (dihydrotestosterone-82)	-0.12	steroid	50-28-2	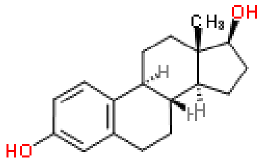
56	17β-hydroxyetiocholan-3-one (dihydrotestosterone-82)	-0.10	steroid	571-22-2	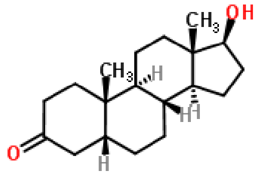
57	17-deoxyestradiol (dihydrotestosterone-82)	-2.13	steroid	53-63-4	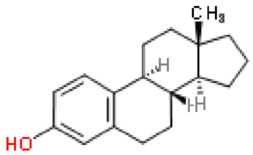

58	Aldrin<br>(N/A)	-2.02	pesticide	309-00-2	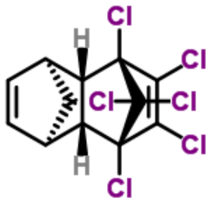
59	Androstenediol<br>(dihydrotestosterone-82)	-0.66	steroid	521-17-5	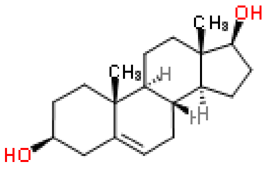
60	Androsterone<br>(dihydrotestosterone-82)	-2.12	steroid	53-41-8	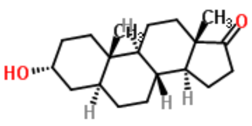
61	Aurin<br>(dihydroxymethoxychlor<br>olefin-83)	-1.70	DDT	603-45-2	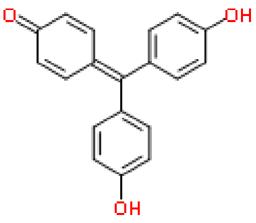
62	Benzophenone<br>(dihydroxymethoxychlor<br>olefin-83)	-2.63	DDT	119-61-9	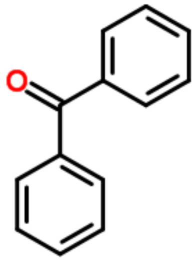

63	bis(*n*-octyl) phthalate (di-*n*-butyl phthalate-88)	-3.28	phthalate	117-84-0	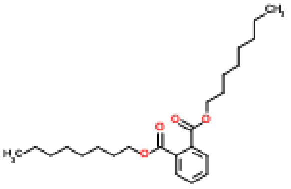
64	Bisphenol A (dihydroxymethoxychlor olefin-83)	-2.39	DDT	80-05-7	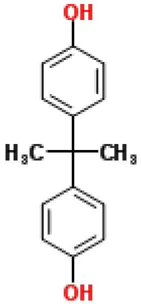
65	Bisphenol B (dihydroxymethoxychlor olefin-83)	-2.09	DDT	77-40-7	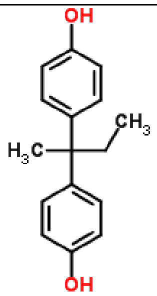
66	Benzylbutylphthalate (di-*n*-butyl phthalate-88)	-2.07	phthalate	85-68-7	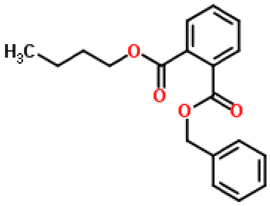
67	β-zearalanol (α-zearalenol-146)	-1.72	phyto	42422-68-4	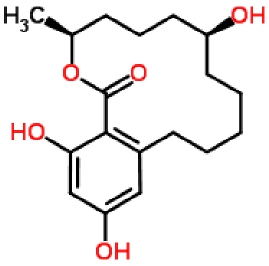

68	β –zearalenol<br>(α-zearalenol-146)	-2.09	phyto	71030-11-0	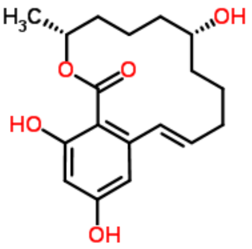
69	Carbaryl<br>(4-hydroxybiphenyl-34)	-3.12	pesticide	63-25-2	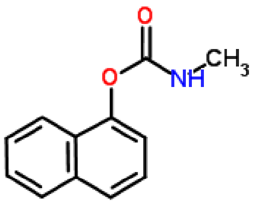
70	Chalcone<br>(6-hydroxyflavanone-51)	-2.32	phyto	94-41-7	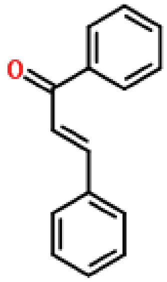
71	Chlordane<br>(*p*-nonylphenol-114)	-1.51	pesticide	57-74-9	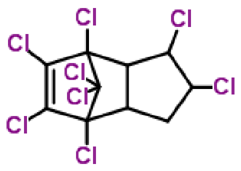
72	Clomiphene A<br>(4-hydroxy-tamoxifen-40)	-1.64	DES	911-45-5	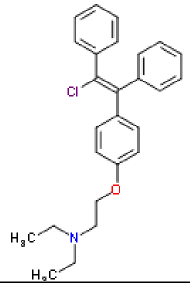

73	Corticosterone (dihydrotestosterone-82)	-1.87	steroid	50-22-6	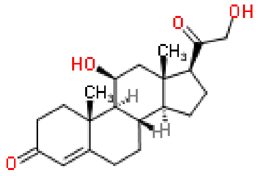
74	Cortisol (dihydrotestosterone-82)	-2.77	steroid	50-23-7	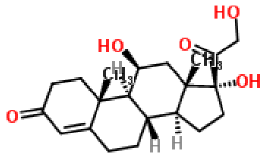
75	cyproterone 17α acetate (dihydrotestosterone-82)	-0.32	steroid	427-51-0	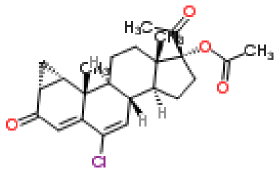
76	Dexamethasone (dihydrotestosterone-82)	-2.42	steroid	50-02-2	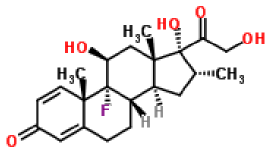
77	5α-dihydrotestosterone benzoate (dihydrotestosterone-82)	0.07	steroid	1057-07-4	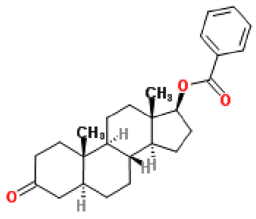

78	Dibutyl adipate<br>(α-zearalenol-146)	-2.73	other	105-99-7	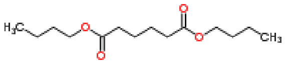
79	Diethyl phthalate<br>(di-*n*-butyl phthalate-88)	-3.44	phthalate	84-66-2	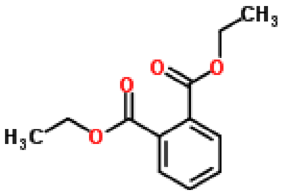
80	Diethylstilbestrol (DES)<br>(4-hydroxy-tamoxifen-40)	-1.66	DES	56-53-1	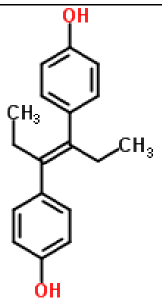
81	1,3-diphenyltetramethyl<br>disiloxane<br>(4-hydroxybiphenyl-34)	-1.95	siloxane	56-33-7	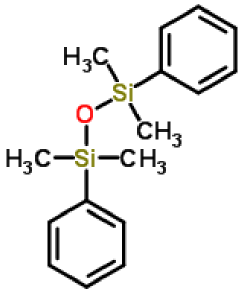
82	Dihydrotestosterone<br>(dihydrotestosterone-82)	2.14	steroid	521-18-6	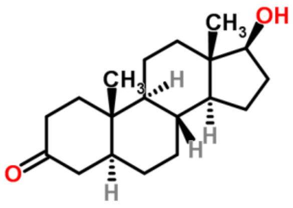

83	dihydroxymethoxychlor olefin (dihydroxymethoxychlor olefin-83)	-1.31	DDT	14868-03-2	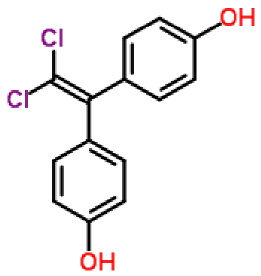
84	di-*i*-butyl phthalate (di-*n*-butyl phthalate-88)	-2.22	phthalate	84-69-5	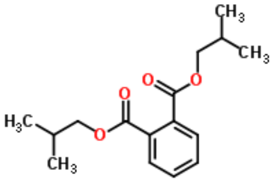
85	diisobutyl adipate (α-zearalenol-146)	-2.84		141-04-8	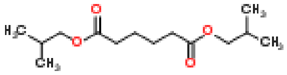
86	diisononyl phthalate (di-*n*-butyl phthalate-88)	-3.56	phthalate	28553-12-0	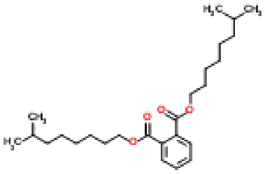
87	Dimethylstilbestrol (4-hydroxy-tamoxifen-40)	-1.66	DES	552-80-7	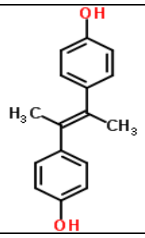
88	di-*n*-butyl phthalate (di-*n*-butyl phthalate-88)	-1.95	phthalate	84-74-2	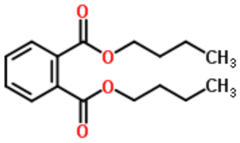

89	β-endosulfan (N/A)	-1.87	pesticide	115-29-7	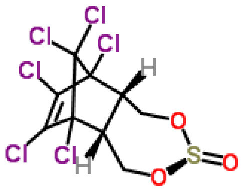
90	Epitestosterone (dihydrotestosterone-82)	-1	steroid	481-30-1	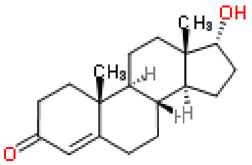
91	(S) equol (6-hydroxyflavanone-51)	-2.39	phyto	531-95-3	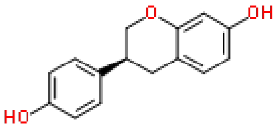
92	Estriol (dihydrotestosterone-82)	-3.15	steroid	50-27-1	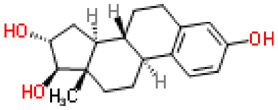
93	Ethyl parathion (4-hydroxybiphenyl-34)	-2.05	pesticide	56-38-2	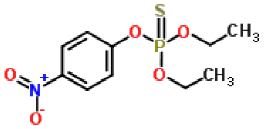

94	Ethynylestradiol (dihydrotestosterone-82)	-1.42	steroid	57-63-6	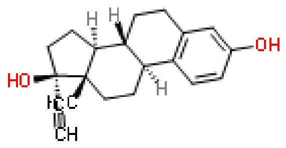
95	Fenpicionil (4-hydroxybiphenyl-34)	-1.61	other	74738-17-3	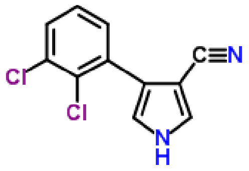
96	(2S) flavanone (6-hydroxyflavanone-51)	-2.25	phyto	487-26-3	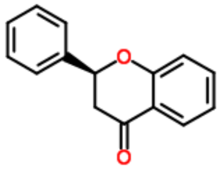
97	Flavone (6-hydroxyflavanone-51)	-2.4	phyto	525-82-6	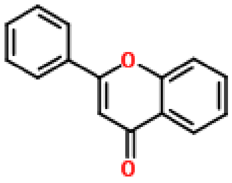
98	Flutamide (2-(4-nitrobenzyl)-1H-isoindole-1,3(2H)-dione-3)	-2.42	flutamide	13311-84-7	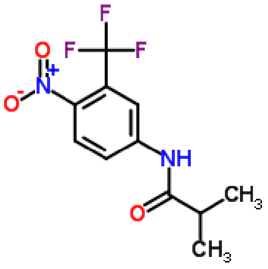

99	Genistein (6-hydroxyflavanone-51)	-2.44	phyto	446-72-0	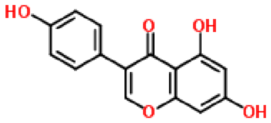
100	Heptachlor (N/A)	-1.64	pesticide	76-44-8	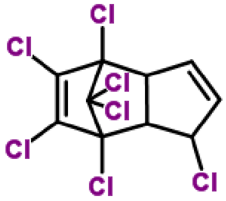
101	HPTE (dihydroxymethoxychlor olefin-83)	-1.47	DDT	2971-36-0	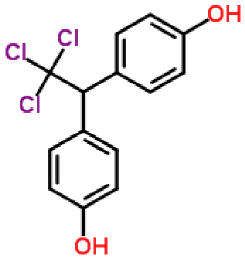
102	Igepal CO-210 (*p*-nonylphenol-114)	-1.78	phenol	26027-38-3	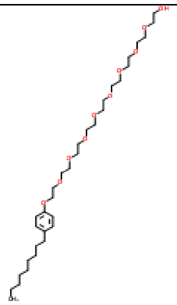
103	Isoeugenol (*p*-nonylphenol-114)	-2.81	phenol	97-54-1	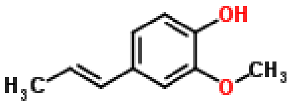

104	Kepone<br>(N/A)	-1.58	pesticide	143-50-0	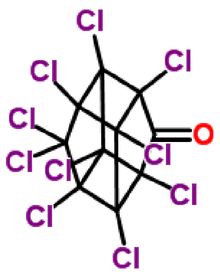
105	γ-Lindane<br>(N/A)	-2.12	pesticide	58-89-9	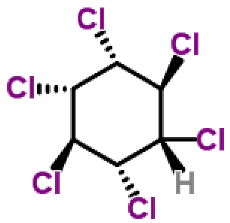
106	Linuron<br>(2-(4-nitrobenzyl)-1H-isoindole-1,3(2H)-dione-3)	-2.25	flutamide	330-55-2	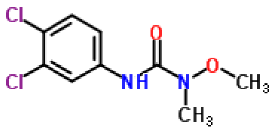
107	Parathion-methyl<br>(4-hydroxybiphenyl-34)	-2.26	pesticide	298-00-0	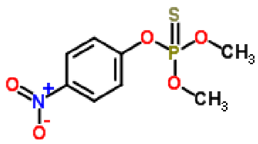
108	Methyltestosterone<br>(dihydrotestosterone-82)	1.28	steroid	58-18-4	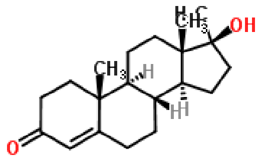

109	Metolachlor (2-(4-nitrobenzyl)-1H-isoindole-1,3(2H)-dione-3)	-2.61	pesticide	51218-45-2	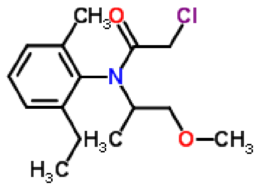
110	Mibolerone (dihydrotestosterone-82)	-1.84	steroid	3704-09-4	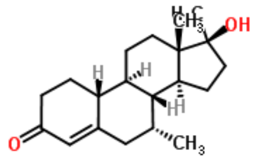
111	monohydroxymethoxychlor olefin (dihydroxymethoxychlor olefin-83)	-1.84	DDT	75938-34-0	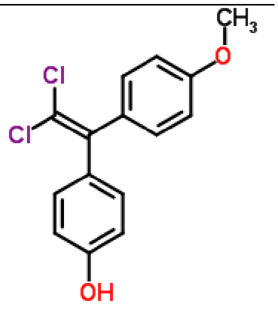
112	monomethyl ether hexestrol (4-hydroxy-tamoxifen-40)	-1.63	DES	13026-26-1	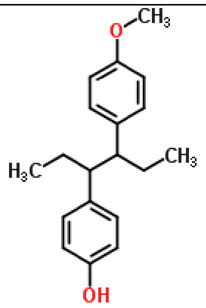
113	Nafoxidine (4-hydroxy-tamoxifen-40)	-1.63	DES	1845-11-0	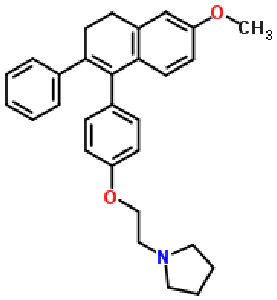

114	*p*-nonylphenol (*p*-nonylphenol-114)	-1.57	phenol	25154-52-3	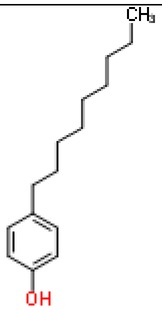
115	nordihydroguaiaretic acid (6-hydroxyflavanone-51)	-2.28	other	500-38-9	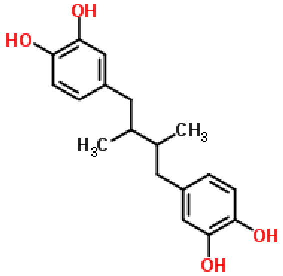
116	Norethindrone (dihydrotestosterone-82)	0.41	steroid	68-22-4	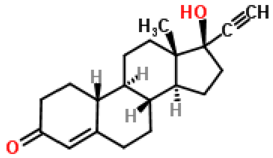
117	Norethynodrel (dihydrotestosterone-82)	-0.7	steroid	68-23-5	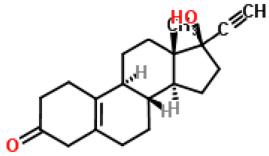
118	*l*-norgestrel (dihydrotestosterone-82)	1.22	steroid	797-63-7	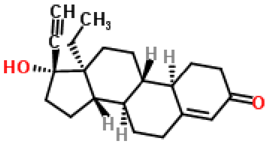

119	n-propyl 4-hydroxybenzoate (*p*-nonylphenol-114)	-3	phenol	94-13-3	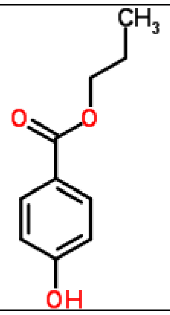
120	*o,p*-DDD (dihydroxymethoxychlor olefin-83)	-1.52	DDT	53-19-0	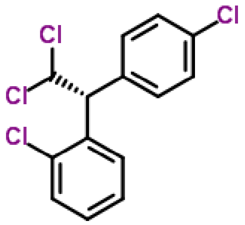
121	*o,p*'-DDE (dihydroxymethoxychlor olefin-83)	-1.81	DDT	3424-82-6	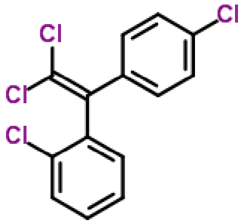
122	*o,p*'-DDT (dihydroxymethoxychlor olefin-83)	-1.69	DDT	789-02-6	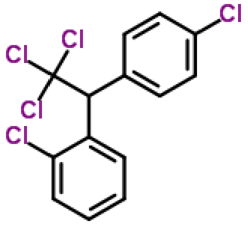
123	*p,p*'-DDD (dihydroxymethoxychlor olefin-83)	-1.7	DDT	72-54-8	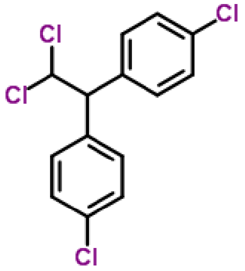

124	*p*,*p*'-DDE (dihydroxymethoxychlor olefin-83)	-1.7	DDT	72-55-9	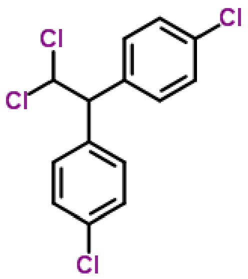
125	*p*,*p*'-DDT (dihydroxymethoxychlor olefin-83)	-1.76	DDT	50-29-3	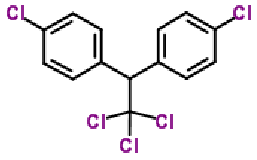
126	*p*,*p*'-methoxychlor olefin (dihydroxymethoxychlor olefin-83)	-2.2	DDT	2132-70-9	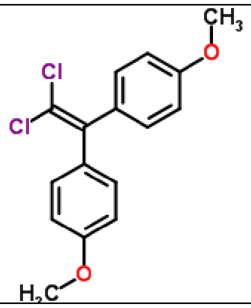
127	*p*',*p*'-methoxychlor (dihydroxymethoxychlor olefin-83)	-1.94	DDT	72-43-5	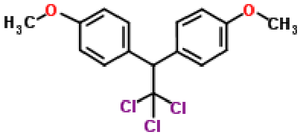
128	*p*-cumyl phenol (dihydroxymethoxychlor olefin-83)	-2.11	DDT	599-64-4	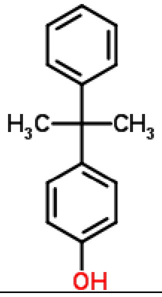

129	Procymidone (2-(4-nitrobenzyl)-1H-isoindole-1,3(2H)-dione-3)	-2.61	pesticide	32809-16-8	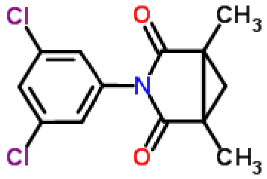
130	Progesterone (dihydrotestosterone-82)	-0.7	steroid	57-83-0	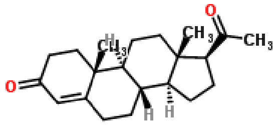
131	Promegestone (dihydrotestosterone-82)	-0.64	steroid	34184-77-5	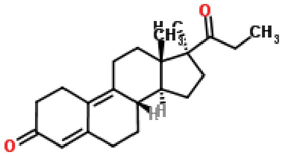
132	Propanil (2-(4-nitrobenzyl)-1H-isoindole-1,3(2H)-dione-3)	-2.22	flutamide	709-98-8	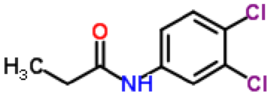
133	R1881 (synthetic androgen) (dihydrotestosterone-82)	2	steroid	965-93-5	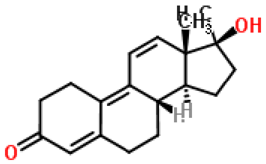

134	Spironolactone (dihydrotestosterone-82)	-0.35	steroid	52-01-7	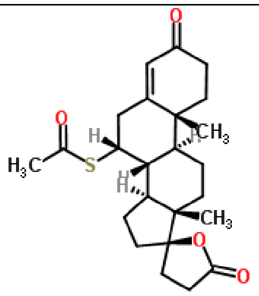
135	Tamoxifen (4-hydroxy-tamoxifen-40)	-1.59	DES	10540-29-1	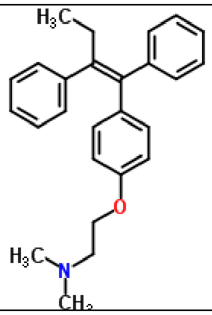
136	testosterone (dihydrotestosterone-82)	1.28	steroid	58-22-0	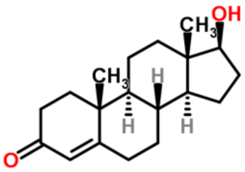
137	testosterone propionate (dihydrotestosterone-82)	-0.79	steroid	57-85-2	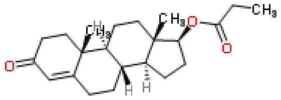
138	testosterone, 11-keto (dihydrotestosterone-82)	0.54	steroid	564-35-2	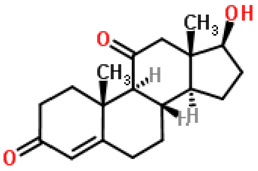

139	trans-4-hydroxystilbene (4-hydroxy-tamoxifen-40)	-2.13	DES	6554-98-9	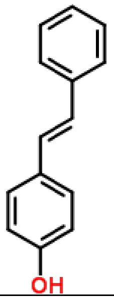
140	trenbolone (dihydrotestosterone-82)	2.05	steroid	10161-33-8	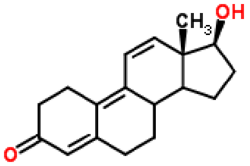
141	triphenyl phosphate (triphenyl phosphate-141)	-1.69	other	115-86-6	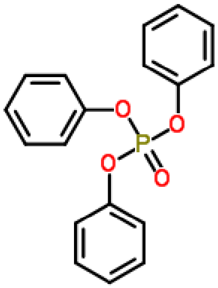
142	triphenylethylene (4-hydroxy-tamoxifen-40)	-1.98	DES	58-72-0	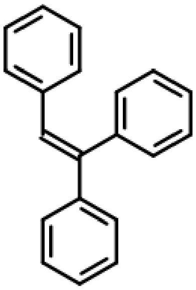
143	triphenylsilanol (triphenyl phosphate-141)l	-2.05	siloxane	791-31-1	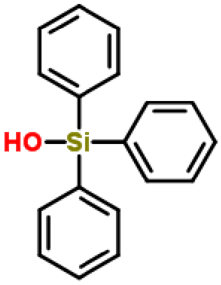

144	Vinclozolin (2-(4-nitrobenzyl)-1H-isoindole-1,3(2H)-dione-3)	-2.5	pesticide	50471-44-8	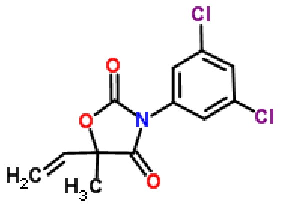
145	zearalanone (α-zearalenol-146)	-2.14	phyto	5975-78-0	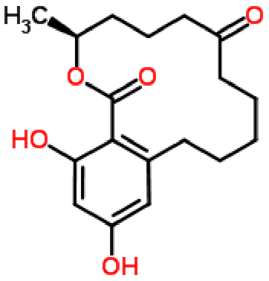
146	α-zearalenol (α-zearalenol-146)	-1.64	phyto	36455-72-8	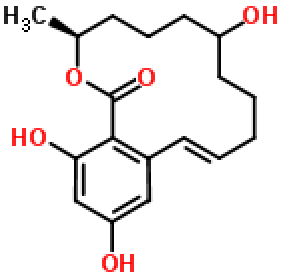

[a] **Structures from http://www.chemspider.com, an online free chemical structure database.**
