# Supplementary material for: Alignment-independent technique for 3D QSAR analysis
Source: J Comput Aided Mol Des. 2016 Mar 30;30:331–45. doi: 10.1007/s10822-016-9909-0 (PMC4833814; doi:10.1007/s10822-016-9909-0)
Supplement: Supplementary file 8 — Supplementary material 8 (PDF 101 kb) [file 10822_2016_9909_MOESM8_ESM.pdf]

For *Journal of Computer-Assisted Molecular Design* Online Resource_8

Alignment Independent Technique for 3D QSAR Analysis

Jon G. Wilkes*[1], Iva B. Stoyanova-Slavova[1], Dan A. Buzatu[1]

1 Division of Systems Biology at the National Center for Toxicological Research, 3900 NCTR Road, Jefferson, AR 72079 USA

*Corresponding Author:
Jon G. Wilkes
NCTR
3900 NCTR Road
Jefferson, AR 72079
T: 1 + (870)543-7108
F: 1+ (870)543-7086
E: jon.wilkes@fda.hhs.gov

ESM_8. MATLab Code used for building PLS Composite 3D-QSDAR Models (Original File Name QSDARExtTest.m)

```
% Reading the binned data from an Excel file
[BinFile]=uigetfile('*.xlsx','Select an Excel file containing the binned data');
[M, bins]=xlsread(BinFile);
BinLabels=bins(2:end);
ACT=M(:,1); % Activity column (endpoint)
MAT=M(:,2:end); % Descriptor matrix
samples=size(ACT,1); % Number of samples (molecules)
ID=(1:samples)';
clear M;

% Reading the external test set from an Excel file
[BinFileExtTst] = uigetfile('*.xlsx','Select an Excel file containing the external test binned data');
[MExtTst, FirstRow] = xlsread(BinFileExtTst);
AETS = MExtTst(:,1); % Ext test set activity column
METS = MExtTst(:,2:end); % Ext test descriptor matrix
ExtPredSz = size(AETS,1);
clear MExtTst;

% Opens a 3D-QSDAR dialog window with the modeling parameters
prompt = {'Number of training/test cycles:','Max Number of LVs:',...
'Number of significant bins:','Test/Training set ratio (0-1):'};
dlg_title = 'PLS parameters input';
num_lines = 1;
def = {'100','5','10','0.2'};
inptval = inputdlg(prompt,dlg_title,num_lines,def);
cycles = cellfun(@str2num,inptval(1,1));
LV = cellfun(@str2num,inptval(2,1));
SignBins = cellfun(@str2num,inptval(3,1));
TstRatio = cellfun(@str2num,inptval(4,1));

% Initialization of the random number generator
s=RandStream('mt19937ar','seed',0);
RandStream.setDefaultStream(s);

TotalR2fit=[];TotalR2pred=[];TotalR2scr=[];TotalExtR2pred=[];TAETSpred=[];

for ValidationCycles = 1:cycles

% Definition of the training and test subsets
[Training, Test] = crossvalind('HoldOut', samples, TstRatio);
IDTR=ID(Training==1); IDTS=ID(Test==1);
ATR=ACT(Training==1); ATS=ACT(Test==1);
MTR=MAT(Training==1,:); MTS=MAT(Test==1,:);

```

```
% Removes columns (descriptors) containing only zeros
% indx contains the indices of the non-zero columns
DescrSum = sum(MTR,1);
indx = find(DescrSum>0);
MTR = MTR(:,indx); MTS = MTS(:,indx); ActBins = BinLabels(indx);

% Removes the zero columns for the ext test set descriptor matrix
METSNoZero=METS(:,indx);

% Predicts the training and the test set activities
[R2fit,R2pred,ATRpred,ATSpred,junk,OrigID] = PLS(MTR,MTS,ATR,ATS,LV,SignBins);

TotSignBins=[];
for I = 1:LV
MSignBins = ActBins(OrigID(:,i)');
TotSignBins = [TotSignBins, MSignBins'];
end

% Performs scrambling - the results are stored in TSRsqpr
ScrATR = ATR(randperm(size(ATR,1)));
[junk,R2scr] = PLS(MTR,MTS,ScrATR,ATS,LV,SignBins);
clear junk;

% Predicts the external test set activities
[junk,ExtR2pred,junk,AETSpred] = PLS(MTR,METSNoZero,ATR,AETS,LV,SignBins);
clear junk;

% Calculates the stats of the models
TotalR2fit = vertcat(TotalR2fit, R2fit);
TotalR2pred = vertcat(TotalR2pred, R2pred);
TotalExtR2pred = vertcat(TotalExtR2pred, ExtR2pred);
TotalR2scr = vertcat(TotalR2scr, R2scr);

ActTR = [IDTR,ATR,ATRpred];
ActTS = [IDTS,ATS,ATSpred];
TAETSpred = vertcat(TAETSpred, AETSpred);

dlmcell('c:\3dsdar\TotSignBins.txt',TotSignBins,'-a','|'); %sign bins
dlmwrite('c:\3dsdar\TotSignBins.txt','----','-append');
dlmwrite('c:\3dsdar\ActTrPred.txt',ActTR,'-append'); % pred act when part of the trn
dlmwrite('c:\3dsdar\ActTsPred.txt',ActTS,'-append'); % pred act when part of the tst
dlmwrite('c:\3dsdar\R2TrPred.txt',R2fit,'-append'); %R2 for the training
dlmwrite('c:\3dsdar\R2TsPred.txt',R2pred,'-append'); %R2 for the test
dlmwrite('c:\3dsdar\R2ExtTsPred.txt',ExtR2pred,'-append'); %R2 for the ext test

% Saves the id generated by the random number generator
```

```
dlmwrite('c:\3dsdar\IDTS.txt', IDTS,'-append','newline','pc');
dlmwrite('c:\3dsdar\IDTS.txt','----','-append','newline','pc');
end

% Averages the predictions for the external test set
Lspace = linspace(1,ExtPredSz,ExtPredSz);
Lspace = repmat(lspace',cycles,1);
TAETSpred = [lspace, TAETSpred];
TAETSpred = sortrows(TAETSpred,1);
TAvgPred = [];
for j = 1:LV
AvgPred = accumarray(TAETSpred(:,1),TAETSpred(:,j+1),[],@mean);
TAvgPred = [TAvgPred, AvgPred];
end

% Calculates the average statistical parameters
AvgR2fit=mean(TotalR2fit); StdR2fit=std(TotalR2fit);
AvgR2pred=mean(TotalR2pred); StdR2pred=std(TotalR2pred);
AvgR2ext=mean(TotalExtR2pred); StdR2ext=std(TotalExtR2pred);
AvgR2scr=mean(TotalR2scr); StdR2scr=std(TotalR2scr);
clear TotalR2fit TotalR2pred TotalR2scr TotalExtR2pred;

dlmwrite('c:\3dsdar\AvgActExtTsPred.txt',TAvgPred,'-append'); % avg act of the ext test
dlmwrite('c:\3dsdar\ActExtTsPred.txt',TAETSpred,'-append'); % ext test activities
dlmwrite('c:\3dsdar\TotAvgR2fit.txt', AvgR2fit,'-append','newline','pc');
dlmwrite('c:\3dsdar\TotStdR2fit.txt', StdR2fit,'-append','newline','pc');
dlmwrite('c:\3dsdar\TotAvgR2pred.txt', AvgR2pred,'-append','newline','pc');
dlmwrite('c:\3dsdar\TotStdR2pred.txt', StdR2pred,'-append','newline','pc');
dlmwrite('c:\3dsdar\TotAvgR2ext.txt', AvgR2ext,'-append','newline','pc');
dlmwrite('c:\3dsdar\TotStdR2ext.txt', StdR2ext,'-append','newline','pc');
dlmwrite('c:\3dsdar\TotAvgR2scr.txt', AvgR2scr,'-append','newline','pc');
dlmwrite('c:\3dsdar\TotStdR2scr.txt', StdR2scr,'-append','newline','pc');
```
